# Primary effusion lymphoma enhancer connectome links super-enhancers to dependency factors

**DOI:** 10.1038/s41467-020-20136-w

**Published:** 2020-12-09

**Authors:** Chong Wang, Luyao Zhang, Liangru Ke, Weiyue Ding, Sizun Jiang, Difei Li, Yohei Narita, Isabella Hou, Jun Liang, Shijun Li, Haipeng Xiao, Eva Gottwein, Kenneth M. Kaye, Mingxiang Teng, Bo Zhao

**Affiliations:** 1 Division of Infectious Disease, Department of Medicine, Brigham and Women’s Hospital and Harvard Medical School, 181 Longwood Avenue, Boston, Massachusetts 02115 USA; 2 Dept. of Medicine, The First Affiliated Hospital, Sun Yat-Sen University, Guangzhou, China; 3 Department of Radiology, Sun Yat-Sen Cancer Center, Sun Yat-Sen University, Guangzhou, China; 4 Department of Microbiology-Immunology, Feinberg School of Medicine, Northwestern University, Chicago, IL 60611 USA; 5 Department of Biostatistics and Bioinformatics, H. Lee Moffitt Cancer Center and Research Institute, Tampa, FL 33612 USA

**Keywords:** Chromatin analysis, Chromatin remodelling, Chromatin structure, Gene silencing

## Abstract

Primary effusion lymphoma (PEL) has a very poor prognosis. To evaluate the contributions of enhancers/promoters interactions to PEL cell growth and survival, here we produce H3K27ac HiChIP datasets in PEL cells. This allows us to generate the PEL enhancer connectome, which links enhancers and promoters in PEL genome-wide. We identify more than 8000 genomic interactions in each PEL cell line. By incorporating HiChIP data with H3K27ac ChIP-seq data, we identify interactions between enhancers/enhancers, enhancers/promoters, and promoters/promoters. HiChIP further links PEL super-enhancers to PEL dependency factors MYC, IRF4, MCL1, CCND2, MDM2, and CFLAR. CRISPR knock out of MEF2C and IRF4 significantly reduces MYC and IRF4 super-enhancer H3K27ac signal. Knock out also reduces MYC and IRF4 expression. CRISPRi perturbation of these super-enhancers by tethering transcription repressors to enhancers significantly reduces target gene expression and reduces PEL cell growth. These data provide insights into PEL molecular pathogenesis.

## Abstract (web summary)

Primary effusion lymphoma (PEL) has a very poor prognosis. Here, the authors perform H3K27ac HiChIP in PEL cells and generate the PEL enhancer connectome, linking enhancers and promoters in PEL, as well as super-enhancers to dependency factors.

## Introduction

Primary effusion lymphoma (PEL) is a rare non-Hodgkin’s lymphoma with very poor prognosis that often occurs in HIV infected people. Even in the highly active antiretroviral therapy (HAART) era, a PEL patient’s median survival is ~6 months, as there is no specific therapy^1^. Kaposi’s sarcoma-associated herpesvirus (KSHV or HHV-8) and Epstein-Barr virus (EBV) are tightly linked to this malignancy. A small subset of KSHV- or EBV-encoded proteins as well as non-coding RNAs and miRNAs play essential roles in PEL pathogenesis. The expression of KSHV genes is important for PEL growth and survival^2–5^. vFLIP (KSHV) and LMP1 (EBV), each activate NF-kB^6,7^. EBV superinfection of KSHV only in PELs increases tumorgenicity in vivo^8^. EBV and KSHV co-infection causes tumor formation in a humanized mouse model, and results in greatly reduced mice survival^9^. EBV can enhance the KSHV genome maintenance in PELs^10^. Analysis of PEL cell genomes revealed that coinfection with EBV is associated with fewer host genomic alterations compared to PELs that are only KSHV infected^11^. PELs that are coinfected with KSHV and EBV differ in gene expression profiles compared with KSHV positive and EBV negative PELs^12^.

PEL cells depend on a group of genes for their continuous proliferation, identified by genome-wide CRISPR screens^13^. These genes include *MYC*, *IRF4*, *CCND2*, *MCL1*, and *CFLAR*, which are important for transcription, cell cycle progression, and survival^13^. The expression of these oncogenes is controlled by enhancers and promoters. Enhancers are marked by unique histone modifications including H3K27ac and H3K4me1, and can be readily identified by chromatin immunoprecipitation followed by deep sequencing (ChIP-seq). However, it is difficult to assign these enhancers to their direct target genes as they can be anywhere in the genome, relative to their direct targets. In EBV transformed lymphoblastoid cell lines (LCLs), chromatin interaction analysis followed by deep paired-end sequencing (ChIA-PET) links enhancer–enhancer, enhancer–promoter, and promoter–promoter interactions genome wide^14^. LCL enhancers hundreds kb upstream loop to the *MYC* promoter to activate *MYC* expression. Enhancers upstream of *IRF4* skip the nearest gene *DUSP22* and interact with *IRF4* that is further away from the enhancer. Some enhancers even interact with genes on other chromosomes^15^. Frequently, important oncogenes are linked to multiple enhancers^14^. However, ChIA-PET is technically challenging as hundreds of millions of cells are needed. The recently developed HiCHIP method can achieve the same goal with much higher efficiency and much less input DNA^16^.

Transcription factors (TFs) and co-factors bind to enhancer DNA to assemble active enhancers. These TFs also recruit histone acetyl transferases (HATs) and histone methyl transferases (HMTs) to modify the histone tails at these enhancers. In different cancers, through altering enhancer DNA sequence, oncogenes can acquire new enhancers through de novo enhancer formation^17^. Viral proteins can form new enhancers by directly binding to enhancer DNA or induce cell TF DNA binding (such as NF-κB) to control key oncogene expression^18–20^, including that of EBV and KSHV proteins^21^. Human papilloma virus integration can function as a super-enhancer (SE) to drive viral oncogene expression^22^. Oncogenic TFs such as NOTCH can assemble SEs to control oncogene expression^23^. SEs can also be acquired by genome amplification^17^. EP300, a HAT, is essential for PEL growth and survival^13^, probably through enhancer activation.

Combinations of different histone modifications mark different genomic regulatory elements^24^. Alterations in histone modifications are often associated with oncogenesis^25^. ChIP-seq and ChIP-on-ChIP analysis of PEL cells identified active and repressed regions in the KSHV genome^26–29^. H3K4me3 and H3K27me3 ChIP-seq analysis of PEL cells identified active promoters and repressed regions genome wide^28,30,31^.

SEs are enhancers with extraordinarily high and broad H3K27ac ChIP-seq peaks compared to average enhancers^21,32^. SEs play critical roles in development and oncogenesis. SEs are co-occupied by many TFs and co-factors, and proposed to form phase-separated nuclear subdomains and are more sensitive to perturbations. SEs frequently target key oncogenes such as MYC^14^. Importantly, SEs are more sensitive to perturbations than typical enhancers^21,24^. Characterization of the SE components may lead to interventions.

Here we generate the PEL enhancer connectomes using H3K27ac HiChIP, linking all PEL enhancers to their direct target genes in both EBV positive and negative PEL cell lines. We then integrate these data with the H3K27ac ChIP-seq data. We find that PEL dependency factors are linked to PEL SEs. CRISPRi validate the functional significance of these PEL SEs in gene expression and cell growth.

## Results

### PEL enhancer interactomes

In order to fit the 2 m long genomic DNA into the tiny nucleus, genomic DNA is packaged in a very complexed yet ordered way. Transcription regulatory elements such as enhancers and silencers are positioned in close proximity to their direct target genes by looping out intervening sequences, even though they can be hundreds of kb away from each other. Next-generation sequencing-based assays now can efficiently identify long-range chromatin interactions to generate connectomes. To link PEL enhancers to their direct target genes, H3K27ac HiChIP was used^16^. PEL cell lines BC1, JSC, BC3, and BCBL1 were chosen for the analyses. BC1 and JSC are EBV positive while BC3 and BCBL1 are EBV negative. PEL cells were first cross-linked with formaldehyde. The DNA was cut with Mbo I. The DNA ends were filled with biotinylated dATP and other unlabeled nucleotides and ligated in situ. H3K27ac ChIP was used to enrich the DNA interactions mediated by H3K27ac. Ligated DNA was captured by avidin beads and paired-end deep sequenced. The sequencing reads were then mapped to the human genome using HiC-Pro^33^. Significant genomic interactions were called using hichipper, normalized by total valid paired interaction reads^34^. H3K27ac HiChIP identified 12,511, 13,821, 8184, and 12,129 significant interactions between H3K27ac peaks in BCBL1, BC3, BC1, and JSC cells.

H3K27ac HiChIP linked multiple enhancers to *PRDM1(BLIMP1)*, *IRF2*, *MYB*, and *MIR21* miRNA (Supplementary Fig. 1). PRDM1 is highly expressed in PEL cells compared with other B cell lymphomas^35^. siRNA silencing of PRDM1 greatly reduced PEL cell growth^36,37^. H3K27ac HiChIP linked the *PRDM1* promoter to >10 enhancer sites ~600 kb upstream and ~22 kb downstream of the *PRDM1* promoter (Supplementary Fig. 1a). IRF2 is an EBV SE target and is essential for the growth of LCLs^38^. IRF2 is important for normal B cell proliferation and antibody production^39^. In PEL cells, extensive looping could be seen between >10 enhancers around the *IRF2* gene body and *IRF2* promoter within genomic loci, up and down stream of the *IRF2* promoter in a 600 kb region (Supplementary Fig. 1b). MYB is a TF critical for B cell development. MYB knock out partially blocks B cell development and reduces B cell survival^40^. H3K27ac HiChIP linked >10 genomic interactions within a ~300 kb window around *MYB* gene. The *MYB* promoter was linked to multiple sites downstream of the *MYB* promoter. These downstream sites were also linked to sites upstream of the *MYB* promoter (Supplementary Fig. 1c). MIR21 is an oncoMIR and is involved in the oncogenesis of multiple cancers^21,41,42^. MIR21 expression is induced by KSHV K15^43^. The *MIR21* promoter was linked to >8 enhancers upstream of the promoter, with some as far as ~120 kb away from the promoter (Supplementary Fig. 1d).

### PEL enhancer/promoter landscapes

H3K27ac ChIP-seq is frequently used to identify enhancers/promoters in various cancers^32,44^. To define the PEL cell enhancer/promoter landscapes, H3K27ac ChIP-seqs were done in BC1, JSC, BC3, and BCBL1 cells, in replicates with input DNA as control. Significant H3K27ac signals were identified by peak calling using MACS2 combined with peak merging across replicates using irreproducible discovery rate (IDR)^45^. Here, 21,166, 26,197, 25,546, and 39,610 significant H3K27ac peaks were identified in BC1, JSC, BC3, and BCBL1 cells, respectively; 56.2%, 50.4%, 49.8%, and 38.3% of the H3K27ac peaks were at promoters (within ± 2 kb of the transcription start site (TSS)) and 43.8%, 49.6%, 50.2%, and 61.7% were in intergenic regions or within gene bodies. Out of 49010 total peaks, 11506 were common for all four cell lines; 208 peaks were common in BC1 and JSC cells but not significant in BC3 and BCBL1 cells; 2247 peaks were common in BC3 and BCBL1 cells but not significant in BC1 and JSC cells (Supplementary Fig. 2a).

To identity TF motifs enriched at PEL enhancers, HOMER^46^ was used. The motifs enriched at the enhancers of PEL cells included E2A, MEF2C, SPI1:IRF, BCL6, RBPJ, NF-kB RELA, and ISRE (Supplementary Fig. 2b). E2A is important for B cell development^47^. MEF2C has been shown recently to be essential for EBV SE activity and MEF2C can affect other TFs binding ESEs^48^. SPI1:IRF composite sites recruit SPI1 and IRF4 heterodimer^49^. Even though SPI1 is not expressed in PEL^50^, SPI1 family member SPIB can also dimerize with IRF4^51^. IRF4 is essential for PEL growth and survival^13^. As examples, H3K27ac ChIP-seq peaks at NF-κB subunit RELA, ASCL1, and p16^INK4A^ loci are shown in Supplementary Fig. 2c. NF-κB plays essential roles in the oncogenesis of various types of cancers and can provide anti-apoptotic signals and promote proliferation^52^. KSHV-encoded vFLIP can activate NF-κB^53^. NF-κB TF family has five subunits including RELA. H3K27ac ChIP-seq signals were abundant at the *RELA* promoter. The ChIP-seq peaks were very broad, ~2 kb wide. Wide peaks were also evident at the 3′ end of *RELA*. H3K27ac peaks were evident at the *ASCL1* promoter in KSHV and EBV coinfected BC1 and JSC cells. H3K27ac peaks were evident near *p16*^*INK4A*^ in EBV negative BC3 and BCBL1 cells. p16^INK4A^ mediated growth arrest can be reverted by LANA^54^. GM12878, an LCL line, also had H3K27ac peaks at the promoters of *RELA* and *ASCL*. No H3K27ac peak was found near *p16*^*INK4A*^ (Supplementary Fig. 2c). GM12878 RELA and IRF4 ChIP-seq peaks were evident at PEL H3K27ac peaks, suggesting the presence of NF-kB and IRF4 motifs in these genomic loci (Supplementary Fig. 2c). TFs recruit EP300/CBP to enhancers/promoters and EP300/CBP acetylate histone lysine residues to mark active enhancers. Therefore, in PEL cells, RELA and IRF4 may recruit histone acetylases to acetylate H3K27 at these sites. The expression level of cell genes close to H3K27ac peaks was significantly higher than in genes lacking H3K27ac peaks or all of the genes in BC1, BC3, and BCBL1 cells by microarray (Supplementary Fig. 2d)^54^.

### PEL enhancer connectomes

H3K27ac HiChIP and ChIP-seq data were incorporated to generate the PEL enhancer regulomes by linking enhancers and promoters. HiChIP identified ~658–2217 (~30–46%) enhancer–enhancer interactions, ~678–2563 (~35–42%) enhancer–promoter interactions, and ~326–1776 (~18–27%) promoter–promoter interactions (Supplementary Fig. 2e).

Pathway analysis of genes common for all four cell lines that had enhancers linked to them found significant enrichment of viral carcinogenesis, transcriptional misregulation in cancer, cell cycle, microRNAs in cancer, influence of Ras and Rho proteins on G1 to S transition, apoptosis, and the PI3K-Akt signaling pathway (Table 1).

**Table 1:** KEGG pathway analysis of PEL enhancer-linked genes.

KEGG pathway	Hits	*P*-value
Viral carcinogenesis	25	6.20E-13
Transcriptional misregulation in cancer	13	8.50E-05
Cell cycle	8	1.00E-02
MicroRNAs in cancer	12	2.20E-02
Influence of Ras and Rho proteins on G1 to S transition	4	2.30E-02
Apoptosis	5	3.10E-02
PI3K-Akt signaling pathway	13	3.40E-02
Induction of apoptosis through DR3 and DR4/5 death receptors	4	4.00E-02

### PEL SEs and their direct genes are essential for PEL growth and survival

**Fig. 1:**
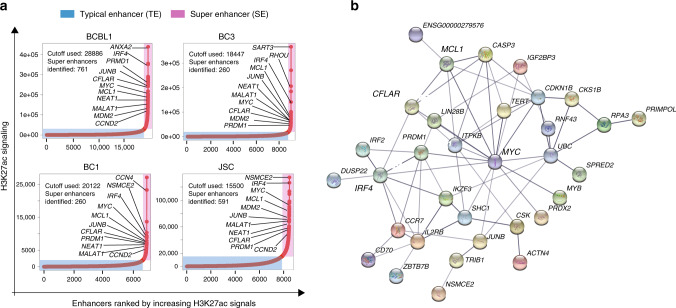
PEL SEs and their linked genes. **a** Enhancers were ranked by their H3K27ac CHIP-seq signals. The inflection point on the plotted curve was then selected as the cutoff to separate SEs from typical enhancers. SEs are indicated in red box. Typical enhancers are indicated in blue box. SEs were linked to their direct target genes by H3K27ac HiChIP. Genes important in B cell biology and oncogenesis are indicated by arrows. **b** SE-linked genes common in four PEL cell lines were analyzed using STRING (https://string-db.org/) with default settings. Gene interaction network formed around MYC is shown.

Enhancers with extraordinarily broad and high H3K27ac ChIP-seq peaks mark the SEs^32^. All enhancers were ranked based on their H3K27ac signals. SEs that had the highest H3K27ac signals were marked in red (Fig. 1a); 761, 260, 260, and 591 SEs were identified in BCBC1, BC3, BC1, and JSC cells. PEL SEs were then linked to their direct targets by H3K27ac HiChIP (Fig. 1a, Supplementary Data 1). String analysis of common PEL SE target genes identified a network centered around the MYC oncogene (Fig. 1b). PEL SEs looped to many additional genes essential for PEL growth and survival identified by genome-wide CRISPR screens, including IRF4, MCL1, and CFLAR (Fig. 1b)^13,55^.

Approximately half of the LCL EBV SEs skip genes immediately adjacent to the enhancers and loop to genes further away in LCLs^21^. The SE looping patterns were also evaluated in PEL cells. Similarly, ~50% of the BCBL1 SEs skipped the nearest gene and looped to genes further away (Supplementary Fig. 2f). These data suggested that ~50% of SEs skipping the nearest gene was common in B cell malignancies.

Looping factor CTCF is a sequence-specific DNA binding protein. When CTCF molecules form a homo-dimer, the distant DNA elements are brought to close proximity and loop out the DNA between two CTCF sites. This allows enhancers to contact other enhancers or promoters^56^. Cohesin subunits SMC1, SMC3, and RAD21 form a ring around the DNA strands to further stabilize the interactions^56^. To evaluate if these looping factors were indeed present at the loops, published PEL CTCF and SMC1 ChIP-seq data were incorporated in our analysis^31^. CTCF and SMC1 ChIP-seq peaks were significantly enriched within the loops linking SEs to their direct target genes comparing the similar sized regions immediately adjacent to the SE loops (Supplementary Fig. 2g, *P* < 2.2e-16, paired Wilcoxon signed-rank test). These data suggested that CTCF and cohesins may mediate the PEL enhancer looping.

### *MYC* SEs are essential for *MYC* expression and PEL cell growth

**Fig. 2:**
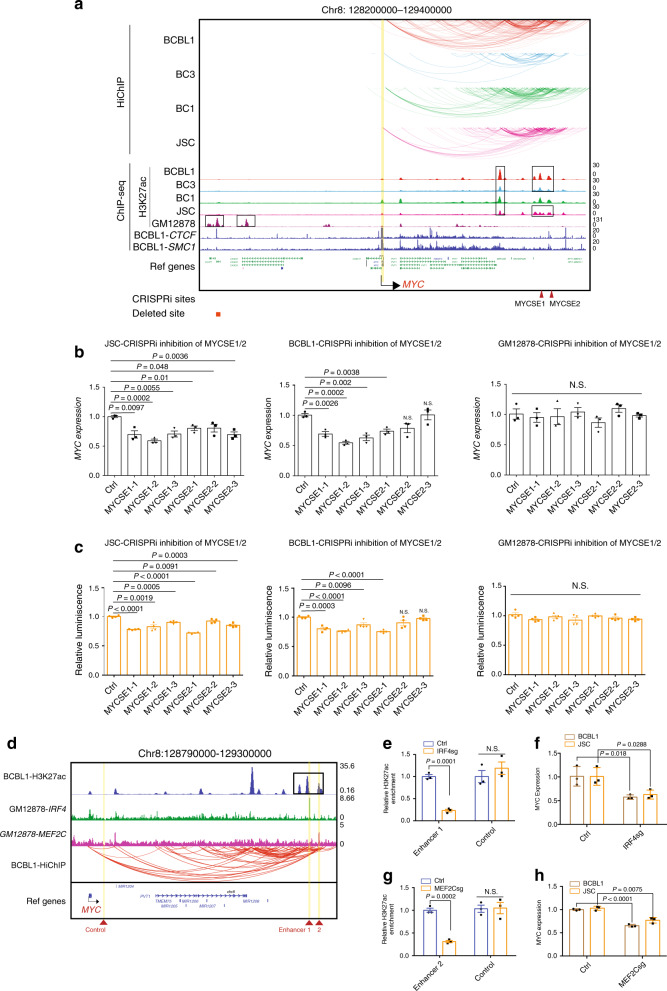
MYC SEs control MYC expression and are essential for PEL cell growth. **a** PEL H3K27ac HiChIP and ChIP-seq tracks, GM12878 H3K27ac, BCBL1 CTCF, and SMC1 ChIP-seq tracks are shown. Black arrow and vertical yellow line indicate *MYC*. Curved line indicates significant HiChIP link. Black boxes indicate SEs. The positions of CRISPRi sgRNAs (MYCSE indicates MYC SE) or dual gRNAs deletion are indicated at the bottom. **b** BCBL1, JSC, or GM12878 cells stably expressing dCAS9-KRAB-MeCP2 were transduced with sgRNA targeting the *MYC* SE or non-targeting control. qRT-PCR was used to quantitate the MYC expression. The levels of control sgRNA-treated cells was set at 1 (*n* = 3, independent experiments). **c** Cell growth following CRISPRi treatment was determined by CellTiter Glo luminescent assay that measures live cell number (*n* = 4, independent experiments). **d** BCBL1 H3K27ac, GM12878 IRF4, MEF2C ChIP-seq, and BCBL1 HiChIP (red curved lines) tracks at the *MYC* locus. *MYC* is indicated by black arrow. *MYC* SE is indicated by black box. Enhancer 1 indicates site of GM12878 IRF4 ChIP-seq peak that overlapped with *MYC* SE and enhancer 2 indicates site of GM12878 MEF2C ChIP-seq peak that overlapped with *MYC* SE. Genomic site near *MYC* promoter that lacked H3K27ac signals was used as control. Yellow lines indicate the positions for qPCR primers used in ChIP assays. **e** IRF4 expression was knock out using CRISPR in BCBL1 cells. SE H3K27ac signals were determined by ChIP-qPCR following CRISPR knock out. The levels of non-targeting control sgRNA-treated cells were set to 1 (*n* = 3, independent experiments). **f** *MYC* expression in BCBL1 and JSC cells following IRF4 knock out. qRT-PCR was used to determine the *MYC* expression levels. The levels of control sgRNA-treated cells were set to 1 (*n* = 3, independent experiments). **g** MEF2C expression was knocked out using CRISPR in BCBL1 cells. SE H3K27ac signals were determined by ChIP-qPCR following CRISPR knock out. The levels of non-targeting control sgRNA-treated cells were set to 1 (*n* = 3, independent experiments). **h** *MYC* expression in BCBL1 and JSC cells following MEF2C knock out. qRT-PCR was used to determine the *MYC* expression levels. The levels of control sgRNA-treated cells were set to 1 (*n* = 3, independent experiments). A two-tailed unpaired *t*-test was used for all statistical analyses. The error bars indicate the SEM for the averages across the all experiments. Source data are provided as a Source Data file.

MYC is essential for oncogenesis and is activated through many different mechanisms in different cancers, such as chromosome translocations, gene amplification, or by SEs^57^. In LCLs, EBV TFs form SEs ~525 and 428 kb upstream of the *MYC* promoter and activate *MYC* expression by looping to the *MYC* promoter^14^. In centroblast B cells and high-grade B cell lymphomas, more H3K27ac signals are at the *MYC* 3′ downstream region^58^. In peripheral blood B cells, mantle cell lymphomas, and small lymphocytic leukemia cells, the H3K27ac peaks are predominantly at the 5′ enhancer regions^58^. In PEL cells, multiple SEs 400–600 kb downstream of *MYC* promoter were found in most PEL lines evaluated (Fig. 2a). No significant H3K27ac signal was found at the GM12878 *MYC* SEs −428 and −525 kb upstream of *MYC* promoter in PELs. The SEs all linked to *MYC* promoter by H3K27ac HiChIP (Fig. 2a). Partial deletion of −525 SE using dual gRNA greatly reduces GM12878 growth and *MYC* expression (Supplementary Fig. 3a-c)^14^. The same deletion did not affect PEL cell growth and *MYC* expression (Supplementary Fig. 3b, c). To determine the functional significance of PEL *MYC* SEs, CRISPRi was used to perturb the enhancers indicated by an arrow (Fig. 2a). JSC and BCBL1 cells were first transduced with lentiviruses expressing an endonuclease-dead CAS9 fused, in frame, to KRAB and MeCP2 transcription repressors^59^. Cells stably expressing the CAS9-repressor fusion protein were transduced with lentiviruses expressing sgRNAs targeting *MYC* SEs ~600 kb down stream of the *MYC* promoter. After puromycin selection, *MYC* expression was determined by qRT-PCR and cell growth was determined by luminescence. Three sgRNAs targeting two regions each within the SE, all significantly reduced *MYC* expression and cell growth in JSC cells (Fig. 2b, c). Most sgRNAs also significantly reduced *MYC* expression and cell growth in BCBL1 cells (Fig. 2c). None of them affected GM12878 *MYC* expression and cell growth. These data suggested that *MYC* SEs are important for PEL *MYC* expression and cell growth. Since IRF4 and MEF2C motifs were present in PEL enhancers, we compared BCBL1 H3K27ac ChIP-seq data and GM12878 IRF4 and MEF2C ChIP-seq data and found GM12878 IRF4 and MEF2C peaks overlapped with BCBL1 H3K27ac peaks within the SE, suggesting the presence of these TF motifs in the PEL SE DNA (Fig. 2d). To determine if IRF4 and MEF2C are important for MYC expression, CRISPR was used to knock out IRF4 and MEF2C (Supplementary Fig. 4). ChIP-qPCR was used to evaluate the effects of CRISPR knock out on *MYC* SE H3K27ac signals. qRT-PCR was used to determine the effects of CRISPR knock out on *MYC* expression. IFR4 knock out significantly reduced H3K27ac signals at *MYC* SE and had no effect on the control region in BCBL1 (Fig. 2e). IRF4 knock out in BCBL1 and JSC cells significantly reduced *MYC* mRNA levels (Fig. 2f). MEF2C knock out significantly reduced H3K27ac signals at *MYC* SE and had no effect on the control region in BCBL1 (Fig. 2g). MEF2C knock out in BCBL1 and JSC cells significantly reduced *MYC* mRNA levels (Fig. 2h). These data indicated that IRF4 and MEF2C were important for *MYC* SE activity and *MYC* expression.

### IRF4 SEs are important for IRF4 expression and PEL cell growth

**Fig. 3:**
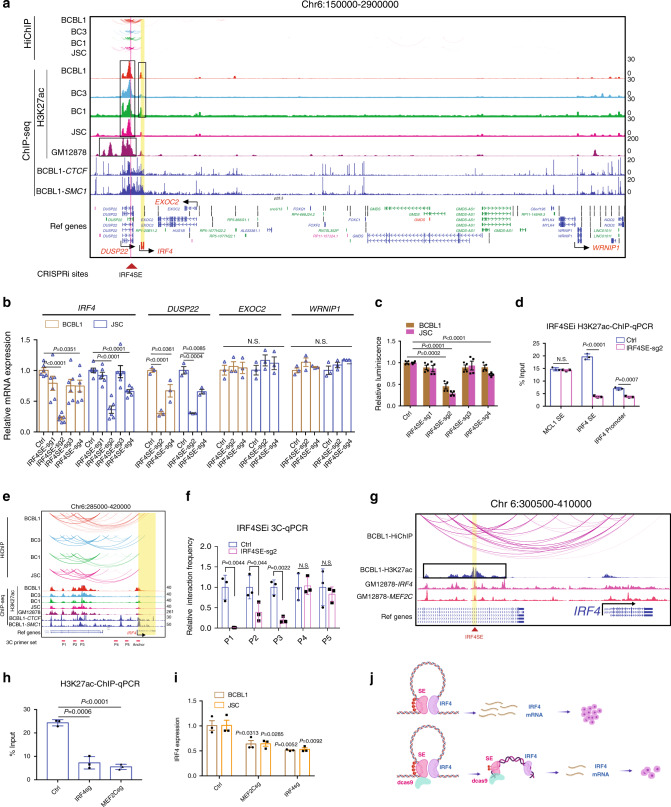
SE control IRF4 expression and PEL cell growth. **a** PEL H3K27ac HiChIP and ChIP-seq tracks, and GM12878 H3K27ac, BCBL1 CTCF, and SMC1 ChIP-seq tracks are shown. Vertical yellow line indicates *IRF4*. Vertical blue line indicates GM12878 IRF4 and MEF2C binding site. Black boxes indicate SEs. Curved line indicates significant HiChIP link. Black arrows indicate neighboring genes with significant H3K27ac ChIP-seq signals. The positions of CRISPRi sgRNAs are indicated at the bottom. **b** RNA expression following CRISPRi inhibition of *IRF4* SE in BCBL1 and JSC cells by qRT-PCR. *EXOC2* and *WRNIP1* not linked by IRF4 SE were used as negative control (*n* = 6, independent experiments for IRF4 mRNA detection, *n* = 3, independent experiments for other genes detection). **c** BCBL1 and JSC cell growth following CRISPRi inhibition of IRF4 SE (*n* = 4, independent experiments. The error bars indicate SEM for the averages across the multiple experiments). **d** H3K27ac ChIP-qPCR at *IRF4* SE and promoter following CRISPRi inhibition of IRF4 SE. *MCL1* SE was used as a negative control (*n* = 3, independent experiments). **e** Positions of anchor primer and other primers used in 3C assay are indicated. **f** CRISPRi or non-targeting control treated cells were fixed and DNAs were cut with Hind III. Diluted DNA ends were ligated. After reverse cross-linking, the DNA was purified and quantitated by qPCR with one primer anchored at IRF4 promoter and the other one in SE or between SE and promoter. The control treated cells were set to 1 (*n* = 3, independent experiments). **g** BCBL1 H3K27ac, GM12878 IRF4, MEF2C ChIP-seq, and BCBL1 HiChIP (red curved lines) tracks at the *IRF4* locus. *IRF4* is indicated by black arrow. *IRF4* SE was boxed in black. Yellow line indicates GM12878 IRF4, MEF2C ChIP-seq peaks overlapped with *IRF4* SE and positions for qPCR primers for the following CRISPR knock out and ChIP-qPCR. **h** MEF2C and IRF4 were knocked down in BCBL1 cells by CRISPR. H3K27ac ChIP-qPCR was done following gene knock out. Control knock out was set to 1 (*n* = 3, independent experiments). **i** *IRF4* expression following MEF2C and IRF4 knock out in BCBL1 and JSC cells by qRT-PCR (*n* = 3, independent experiments). **j** Graphic model of *IRF4* SE silencing by CRISPRi decreased IRF4 SE H3K27ac signals and *IRF4* SE–promoter interactions, leading to suppressed IRF4 expression and reduced cell growth. A two-tailed unpaired *t*-test was used for statistical analysis. The error bars indicate the SEM for the averages across the multiple experiments. Source data are provided as a Source Data file.

IRF4 is essential for both LCLs and PELs^13,38^. SEs were present at the *IRF4* promoter in BC1, BC3, and BCBL1 cells. SEs were also present in the neighboring *DUSP22* gene body (Fig. 3a). Importantly, these SEs ~45 kb upstream of *IRF4* linked to *IRF4* TSS in all four PEL cell lines by HiChIP (Fig. 3a). CRISPRi was used to evaluate the importance of *IRF4* SE within the DUSP22 gene body on IRF4 expression and PEL cell growth. Four different sgRNAs targeting the SE were packaged into lentiviruses and transduced into BCBL1 and JSC cells stably expressing dCAS9-KRAB-MeCP2. After selection, *IRF4* expression was determined by qRT-PCR. Cell growth was determined by luminescence. Two of the sgRNAs significantly repressed *IRF4* expression in both cell lines (Fig. 3b). The same sgRNA also repressed *DUSP22* expression. These sgRNAs had no effect on the expression of *EXOC2* and *WRNIP1*, two genes that are nearby but lacked a HiChIP link to SEs and had H3K27ac peaks at their promoters (Fig. 3b). The same sgRNAs also reduced PEL cell growth in three out of four assays (Fig. 3c). ChIP-qPCR was used to determine the effects of CRISPRi on H3K27ac signals at the *IRF4* SE and promoter. CRISPRi significantly reduced H3K27ac signals at the *IRF4* SE and promoter without affecting the H3K27ac signals at the *MCL1* SE (Fig. 3d). We also tested if CRISPRi affected *IRF4* SE looping to the *IRF4* promoter. Chromatin conformation capture followed by qPCR was used to determine the interactions between SE and IRF4 promoter in the presence or absence of CRISPRi. Cells were first cross-linked and DNAs were cut by Hind III. After dilution, DNA ends were ligated. The purified reverse cross-linked DNAs were quantitated using one primer anchored near the *IRF4* promoter and 5 reverse primers spanning the ~45 kb regions (Fig. 3e). CRISPRi significantly inhibited the interactions between the three primers within the *IRF4* SE and *IRF4* promoter (Fig. 3f). CRISPRi had little effect on interactions between the IRF4 promoter and regions between the SE and promoter (Fig. 3f). GM12878 IRF4 and MEF2C ChIP-seq peaks were also present in PEL *IRF4* SE sites (vertical blue line, Fig. 3a, g). The effects of IRF4 and MEF2C CRISPR knock out on *IRF4* SE H3K27ac signal and IRF4 expression were also evaluated. IRF4 and MEF2C knock out significantly reduced IRF4 SE H3K27ac signals in BCBL1 cells (Fig. 3h). IRF4 and MEF2C knock out also significantly reduced *IRF4* gene expression in both BCBL1 and JSC cells (Fig. 3i). These data suggested that in PEL cells, *IRF4* SE within the DUSP22 gene body can loop to the IRF4 promoter, activating its expression and ensuring PEL cell growth and survival (Fig. 3j).

To further evaluate the contribution of *IRF4* SE and IRF4 to the PEL enhancer landscape, H3K27ac ChIP-seqs were done in BC1 cells treated with control CRISPRi sgRNA or sgRNA targeting *IRF4* SE. *IRF4* SE sgRNA2 reduced H3K27ac signals at the *IRF4* SE and promoter (Supplementary Fig. 4b). In control sgRNA-treated cells, 165 SEs were identified. In *IRF4* SE sgRNA2-treated cells, only 66 SEs were identified (Supplementary Fig. 4c, d). These data suggested that *IRF4* SE and IRF4 can globally affect the SE formation in PEL cells.

### *MCL1*, *CCND2*, and *MDM2* SEs are important for their expression and PEL cell growth

**Fig. 4:**
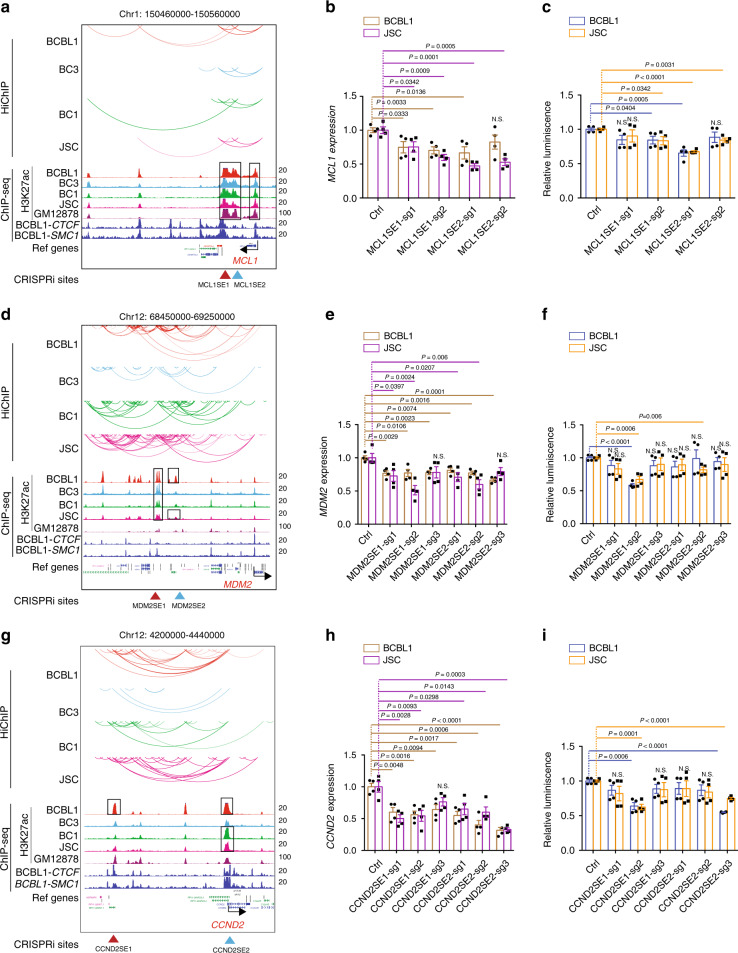
SEs control MCL1, MDM2, and CCND2 expression and PEL cell growth. **a** H3K27ac HiChIP linked SEs to *MCL1*. H3K27ac HiChIP links between SEs and target genes are shown by curved lines on the top. H3K27ac ChIP-seq tracks are shown under the links. BCBL1 CTCF and SMC1 tracks are also shown. SEs are marked by black boxes. **b** CRISPRi targeting SE significantly reduced *MCL1* gene expression, by qRT-PCR. The levels of control sgRNA-treated cells were set to 1 (*n* = 4, independent experiments). **c** CRISPRi targeting *MCL1* SE inhibits cell growth. PEL cell growth following CRISPRi treatment was measured by CellTiter Glo luminescent assay that measures live cell number (*n* = 4, independent experiments). **d** H3K27ac HiChIP linked SEs to *MDM2*. **e** CRISPRi targeting SEs significantly reduced *MDM2* gene expression (*n* = 4, independent experiments). **f** CRISPRi targeting *MDM2* SE inhibits cell growth (*n* = 4, independent experiments). **g** H3K27ac HiChIP linked SEs to *CCND2*. **h** CRISPRi targeting *CCND2* SE significantly reduced *CCND2* expression (*n* = 4, independent experiments). **i** CRISPRi targeting *CCND2* SE inhibits cell growth (*n* = 4, independent experiments). A two-tailed unpaired *t*-test was used for statistical analysis. The error bars indicate the SEM for the averages across the multiple experiments. Source data are provided as a Source Data file.

SEs were present at the *MCL1* gene body and its 3′ region in all PEL cell lines. Major H3K27ac peaks are looped to the *MCL1* promoter region in all PEL cell lines (Fig. 4a). To determine if *MCL1* SEs are functionally important for *MCL1* expression, CRISPRi was used. Seven out eight sgRNAs significantly reduced *MCL1* expression (Fig. 4b). Five out of eight sgRNAs also inhibited PEL cell growth (Fig. 4c). SEs ~450 kb upstream of *MDM2* looped to *MDM2* or its nearby site that further looped to *MDM2* in BCBL1, BC3, and JSC cells, skipping some genes between SEs and *MDM2*. Additional SEs were present near *MDM2* SEs but they did not loop to the *MDM2* gene (Fig. 4d). Ten out of 12 sgRNAs significantly reduced *MMD2* expression by CRISPRi (Fig. 4e). Three out of 12 of the sgRNAs also inhibited PEL cell growth (Fig. 4f). SEs were present at the *CCND2* promoter in BCBL1, BC1, and JSC cells. A cluster of enhancers ~160 kb upstream of *CCND2* also looped to *CCND2* TSS in BCBL1, BC1, and JSC cells (Fig. 4g). All 12 sgRNAs significantly reduced *CCND2* expression by CRISPRi (Fig. 4h). Four out 12 of the sgRNAs also inhibited PEL cell growth (Fig. 4i). CRISPRi had no effects on the expression of neighboring genes (Supplementary Fig. 5a, b)

SEs were also present in *CFLAR* TSS/first intron, *TYRO3* loci in all or some PEL cell lines^60^, and long non-coding RNAs, such as *NEAT1* and *MALAT1* (Supplementary Fig. 6).

### PEL SEs are sensitive to THZ1 and JQ1 perturbations

**Fig. 5:**
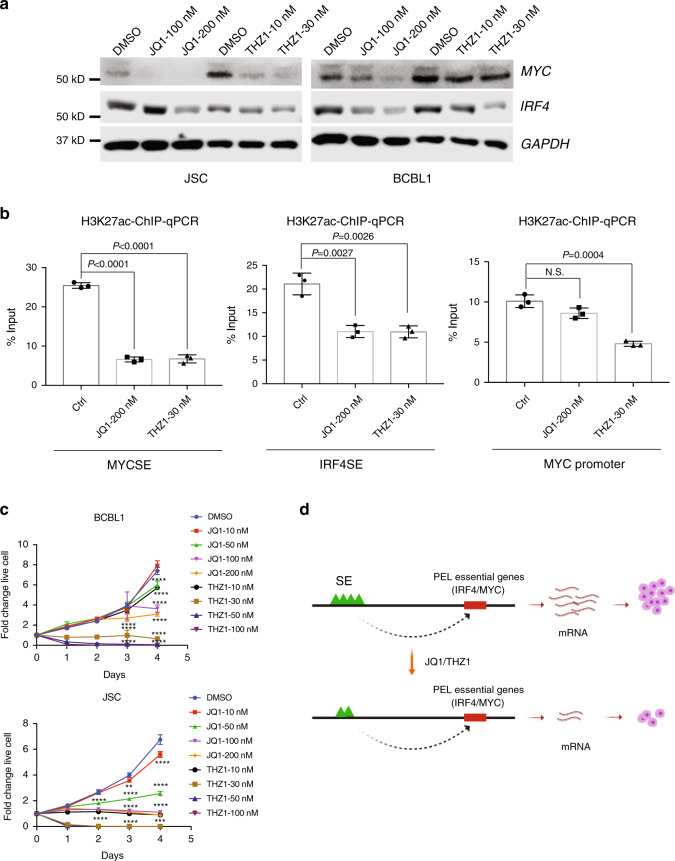
PEL SEs are sensitive to THZ1 and JQ1 perturbations. **a** MYC and IRF4 expression in BCBL1 and JSC cells treated with increasing doses of JQ1 and THZ1 or vehicle for 2 days was determined by immune blotting. GAPDH was used as loading control (*n* = 3, independent experiments with similar results). **b** BCBL1 H3K27ac ChIP-qPCRs signals at *MYC* SE, *IRF4* SE, and *MYC* promoter following JQ1 and THZ1 or vehicle treatment for 2 days (*n* = 3, independent experiments). A two-tailed unpaired *t*-test was used for statistical analysis. **c** BCBL1 and JSC cell growth following treatment with different doses of JQ1 or THZ1, DMSO was used as control. Cell growth was measured by luminescent cell viability assay (*n* = 4, independent experiments). Two-way analysis of variance (ANOVA) followed by Bonferroni multiple comparison analysis was used for analysis. **d** Graphic model of THZ1 and JQ1 treatment decreased PEL SE H3K27ac signals, which further downregulated SE-linked essential genesʼ (IRF4 and MYC) expression and inhibited cell growth. The error bars indicate the SEM for the averages across the multiple experiments. Source data are provided as a Source Data file.

CDK7 inhibitor THZ1 blocks the phosphorylation of RNA polymerase II C-terminal domains and prevents POL II from active transcription^61^. JQ1 prevents BRD4 from binding to acetylated lysines^44^. Transcription co-factors form phase-separated condensates at SEs^62^. JQ1 can effectively block PEL cell growth and prevent KSHV genome looping^63,64^. JQ1 also reduces enhancer H3K27ac^65^. To evaluate the effects of JQ1 and THZ1 treatment on PEL SE activities, BCBL1 and JSC cells were treated with JQ1, THZ1, or vehicle for 48 h. MYC and IRF4 protein levels were decreased following the treatment by western blot (Fig. 5a). JQ1 and THZ1 treatment also significantly reduced H3K27ac signals at the MYC and IRF4 SEs by ChIP-qPCR (Fig. 5b). THZ1 treatment also significantly reduced H3K27ac signals at the MYC promoter (Fig. 5b). JQ1 and THZ1 treatment also significantly reduced PEL cell growth (Fig. 5c), supporting the notion that SEs are susceptible to perturbations (Fig. 5d).

### Enhancer landscapes of EBV and KSHV in PEL cells

To evaluate the epigenetic landscapes of the viral genome in PEL cells, ChIP-seq reads were also mapped to the viral genomes. The EBV genome had major H3K27ac peaks at the non-coding RNA EBER, Q promoter (Qp) which drives EBNA1 expression, and microRNA (Supplementary Fig. 7a). Minor H3K27ac peaks present origin or replication (ori-P), LMP2A, and LMP1, which is consistent with low levels of LMP2A expression in PEL cells. PEL H3K27ac ChIP-seq reads were also mapped to the KSHV and EBV genome. Similar to previous studies^27,28^, significant H3K27ac peaks were found at 20, 80, and 120–140 kb of the KSHV genome (Supplementary Fig. 7b).

## Discussion

H3K4me1 and H3K27ac mark active enhancers and promoters while H3K4me3 marks active promoters. KSHV LANA binds to host chromatin and associates with the H3K4 methyltransferase hSET1 complex^28^. H3K4me3 ChIP-seq identified active promoters in BCBL1 cells^28^. Here we used H3K27ac ChIP-seq to map the active enhancers and promoters in PEL cells. We identified >10,000 significant enhancer sites that were located in the intergenic region or introns for BCBL1, BC3, BC1, and JSC cells. However, H3K27ac ChIP-seq cannot assign each enhancer to its direct target gene. Therefore, we used H3K27ac HiChIP to link these enhancers to their direct target genes, and generated enhancer connectomes of four PEL cell lines. We identified >8000 enhancer–promoter, enhancer–enhancer, and promoter–promoter interactions in these cell lines. The connectome linked SEs to dependency factors MYC, IRF4, MCL, MDM2, and CCND2^66^.

Transcription is regulated efficiently by looping out genomic regions between active enhancers and promoters. Chromatin conformation capture-based assays interrogate the genomic interactions between remote genomic loci. Here, we used H3K27ac HiChIP to define the enhancer connectome of PEL cells, linking enhancers to their direct target genes genome-wide. We found that numerous enhancers loop to their direct target genes. These interactions were shown to be functional in PEL cells as CRISPRi tethering of transcription repressors to enhancers significantly reduced the expression of enhancer-associated genes.

Transcription profiling of PEL cells showed a resemblance of gene expression patterns similar to plasma cells. PRDM1 plays a critical role in plasma cell differentiation, probably through manipulating MYC expression. Our motif analyses found significant enrichment of the PRDM1 motif in PEL H3K27ac peaks^67^. BCL6, on the other hand, is a marker of germinal center B cells^68^. Surprisingly, the BCL6 motif was also enriched in PEL H3K27ac peaks. These data suggested that PEL cells are different from classical plasma cells. Since germinal center B cells are rapidly growing cells, BCL6 may provide a proliferation signal to ensure PEL cell proliferation.

SEs are more sensitive to perturbations than typical enhancers and are ideal therapeutic targets. Therefore, full characterization of SE may lead to development of cancer therapies. Similar to SEs in many other cancers, PEL SEs were linked to key oncogenes such as MYC. MYC is also under the control of SEs in other types of B cell lymphomas, such as mantle cell lymphoma and LCLs^14,57^. In PEL cells, MYC SEs were downstream from MYC TSS, similar to MYC SEs in centroblasts and high-grade B lymphoma cells^23,58^. GM12878 MYC SEs are co-occupied by many B cell TFs such as ETS1, STAT5, IRF4, NFAT, EBF, E2A, and SPI1; all essential EBV TFs; and co-factors^21^. These cell TFs may also be important for PEL MYC SE formation. IRF4 is essential for both GM12878 and PEL growth and survival^13,38^ and binds to PEL enhancers^69^. IRF4 is also controlled by SEs in both GM12878 and PEL cells. In B cells, IRF4 can dimerize with SPI1 to bind to EICE sites^49^. IRF4 may contribute to PEL cell growth and survival by regulating other essential genes’ expression. This is supported by the enrichment of EICE sites in PEL enhancers. CCND2 is critical for cell cycle progression. SEs were found upstream of *CCND2* TSS in BCBL1, BC1, and JSC cells. Additional enhancers were also evident ~180 kb upstream of CCND2 TSS and looped to *CCND2*. MCL1 and CFLAR are both anti-apoptotic that were both linked to SEs in both GM12878 and all PEL lines tested. *MDM2* had multiple SEs ~450 kb upstream of TSS. HiChIP linked the SEs to *MDM2* TSS or neighboring enhancers in BCBL1, BC3, and JSC cells. MDM2 can degrade the tumor suppressor TP53^70^. PEL SEs were also linked to microRNAs and lncRNAs. Oncomir MIR21 is linked to SEs in both PEL and GM12878^14^. In LCLs, MIR155 is also linked to SE. But in PEL cells, no SE was linked to MIR155, consistent with the lack of MIR155 expression in PELs and the presence of a KSHV mimic of MIR155^71^. LncRNA NEAT1 and MALAT1 play important roles in oncogenesis, in PEL cells, the both were linked to SEs.

BRD4 and CDK7 are essential components of SEs in various cancers. BRD4 and CDK7 inhibitors JQ1 and THZ1 can kill many different cancer cells^21^. JQ1 can effectively induce PEL cell growth arrest, apoptosis, and senescence^63^. Additional targets can be identified by further analyses to fully characterize the profiles of TFs that bind to PEL SEs and what co-factors are recruited by these TFs. It is also not clear how these SEs loop to their target gene and how known looping factors CTCF and cohesins contribute to the long-range genomic interactions. Comprehensive understanding of the PEL SEs may identify therapeutic targets.

## Methods

### Cell culture

PEL cells were cultured in RPMI1640 media supplemented with 20% fetal calf serum (Gibco), 100 unit/mL streptomycin, and 100 mg/mL penicillin (Gibco). HEK293T cells purchased from ATCC were cultured in Dulbecco modified Eagle medium supplemented with 10% fetal calf serum (Gibco), 100 unit/mL streptomycin, and 100 mg/mL penicillin. All the cells were maintained at 37 °C in a 5% CO_2_ humidified chamber. Cells were confirmed to be mycoplasma negative.

### Antibodies

Antibodies used in this study are: H3K27Ac (Abcam, Cat: ab4729, dilution: 1:2000), GAPDH (Abcam, Cat: ab9485, dilution: 1:5000), MYC (Cell Signaling Technology, Cat: 13987, dilution: 1:1000), MEF2C (Cell Signaling Technology, Cat: 5030, dilution: 1:500), IRF4 (Santa Cruze, Cat: sc-377383, dilution: 1:500), and CAS9 (Thermo Fisher, Cat: MA1-201, dilution: 1:1000).

### ChIP-seq and HiChIP

ChIP-seq was done following^19^. PEL cell H3K27Ac HiChIP libraries were prepared following^16^. In brief, 15 million cells were cross-linked with 1% formaldehyde and lysed with Hi-C lysis buffer (10 mM Tris-HCl pH 7.5, 10 mM NaCl, 0.2% NP-40, 1X Roche protease inhibitors). Cross-linked DNA was digested by 375 U Mbo I at 37 °C for 2 h. DNA ends were filled in with biotin-dATP (Thermo Fisher 19524016), dCTP, dGTP, and dTTP mix, and DNA Polymerase I, Klenow fragment (NEB, M0210) with shaking at 37 °C for 1 h. T4 DNA Ligase was used for proximity DNA ligation at 4 °C overnight. After ligation, DNA was sonicated using a Covaris M220. Fragmented DNA were then diluted 10 times with ChIP dilution buffer (0.01% SDS, 1.1% Triton X-100, 1.2 mM EDTA, 16.7 mM Tris-HCl pH 7.5, 167 mM NaCl) and samples precleared with Protein A Dynabeads at 4 °C for 1 h. DNA–protein complexes were captured with 8 μL H3K27Ac antibody at 4 °C overnight. DNA–protein complexes were captured with Protein A beads with rotation at 4 °C for 2 h. Beads were washed and DNA were eluted twice with 150 μL of freshly prepared DNA Elution Buffer (50 mM sodium bicarbonate pH 8.0, 1% SDS). The eluted DNA was reverse cross-linked. DNA was purified with a PCR purification Kit (Qiagen). Biotin dATP labeled DNA was captured with 5 μL Streptavidin C-1 beads (Thermo Fisher, #65001) and DNA was then transposed with 2.5 μL of Tn5. Beads were then washed. After the final wash, beads were re-suspended with 23 μL ddH2O, 25 μL 2X Phusion HF (New England Biosciences), and 1 μL of each of Nextera forward primer (Ad1_noMX) and Nextera reverse primer (Ad2.X) at 12.5 μM. PCR was run at: (1) 72 °C for 5 min, (2) 98 °C for 1 min, (3) 98 °C for 15 s, (4) 63 °C for 30 s, and (5) repeat steps 1–4 for a total of 8 cycles, and final extension at 72 °C for 1 min. After PCR amplification, a two-sided size selection with Ampure XP beads was performed for DNA size selection and purification. Samples were then sequenced on the Illumina Nextseq platform (2 × 75 bp).

### CRISPR knock out

BCBL1 was transduced with CAS9 expressing lentivirus followed by Blasticidin selection. sgRNAs from the Brunello library were used to knock out gene of interest. sgRNAs were packaged into lentiviruses. Lentiviruses were prepared by transfecting HEK293T cells with pCMV-VSVG (# 8454; Addgene), psPAX2 (#12260; Addgene), and pLenti-Guide-puro plasmid expressing gRNA with TransIT-LT1 transfection reagent (Mirus) according to the manufacturer’s instructions. Viruses were used to transduce BCBL1 cells; 48 h after infection, cells were selected by 3 μg/mL Puromycin. Cells were then allowed to grow for another 3 days.

### CRISPRi repression

BCBL1 and JSC stably expressing dCAS9-KRAB-MeCP2 fusion proteins were selected by blasticidin. Cells were transduced by lentiviruses expressing sgRNA. Cells were selected with puromycin and then allowed to grow for 3 days.

### Cell growth assay

CellTiter-Glo^®^ Luminescent Cell Viability Assay (Promega) was used to determine the relative number of viable cells in each condition.

### qRT-PCR

Total mRNAs were extracted using PureLink RNA mini kit (Life Technologies). Two hundred nanogram of mRNA was used as template for reverse transcription with iScript™ Reverse Transcription Supermix (Bio-rad). cDNAs were then amplified on an CFX96 Touch real-time PCR detection system (Bio-Rad), and SYBG Green (Thermo Fisher) was used to detect cDNA amplification. GAPDH was used to normalize gene expression. RNA relative expression was calculated using the 2 ΔΔCT method. The value for the cells transduced with non-targeting sgRNA was set to 1.

### ChIP-qPCR

Ten million cells were fixed with 1% formaldehyde. The cells were then lysed and lysates were sonicated with bioruptor (Diagenode) with 30 s on, 30 s off for 45 cycles. Sonicated chromatin was diluted with ChIP dilution buffer and incubated with H3K27ac or control antibodies. Protein–DNA complexes were precipitated with protein A beads. After precipitation beads were washed extensively and eluted protein–DNA complexes were reverse cross-linked. DNA was purified by using QIAquick Spin columns (Qiagen). qPCR was used to quantify the DNA from ChIP assay and normalize it to the percent of input DNA. Primers used in this study are provided in Supplementary Data 2.

### Microarray data and processing

Gene expression microarray data were download from GEO (GSE1880) for BC1, BC3, and BCBL1 cell lines, followed by normalization using RMA^72^. Normalized signals of probes targeting the same genes were averaged for each gene. Genes were further categorized into two groups for expression comparison: H3K27Ac peaks within 20 kb upstream of genes’ transcription start site or no peaks otherwise.

### ChIP-seq and HiChIP data analysis

FastQC (https://www.bioinformatics.babraham.ac.uk/projects/fastqc) was performed on both ChIP-seq and HiChIP sequencing reads to ensure sequencing experiments have no considerable flaws such as heavy GC bias and PCR artifacts. ChIP-seq reads were aligned to human (hg19) and EBV (Akata) genomes using Bowtie2 v2.2.3^73^ under default settings except parameter −*k* was set to 1. Read mappability rate ranges from 94 to 98% across ChIP-seq samples. ChIP-seq peaks were called using MACS v2.1.0^74^ (default settings except reporting criteria was set as FDR < = 0.99) on each replicated sample followed by IDR analysis (v2.0.3), default settings except reporting criteria was set as IDR < = 0.02, which is suggested by ENCODE consortium^75,76^ to generate reproducible peaks between replicates. Peaks located in blacklist regions^77^ were excluded in analysis. The HOMER program^46^ was then used to detected motifs in ChIP-seq peaks. Genome-wide ChIP-seq coverage were normalized with size factors which were determined using DiffBind^78^ combined with DESeq2^79^. SEs were called with ROSE v1.0.0 under default settings^32^ using IDR-reported H3K27ac peaks. HiChIP paired-end reads (17–27 million reads for each sample) were mapped using HiC-Pro v2.11.1^33^ (default settings with LIGATION_SITE set as GATCGATC for Mbo I) and significant loops identified with hichipper v0.7.5^34^ (default settings except parameter—skip-diffloop was set). In detail, HiChIP experiments are performed with Mbo I, which can introduce a biasness to the fragment sizes due to non-uniform distribution of cut sites. hichipper performs a background correction based on the non-uniform distribution of restriction fragments, to better infer anchors and loops. Quality control was performed by ensuring a high percentage of reads were mapped (>90%), within anchors (>65%) and supporting valid interactions (25–35%). Mapped reads were also visually inspected on the WashU genome browser^80^ to ensure significant enrichment when compared to H3K27ac ChIP-seq reads, general uniformity between replicates, and good signal-to-noise ratio, which are indicative of a successful ChIP experiment. Replicates samples were then merged and loops were identified by (1) merging anchors within 1.5 kb of each other, (2) removing loops that are <5 kb in length. Loops scores were further normalized by total number of valid interaction read pairs in each cell line. A minimum of three normalized read pairs were used to filter strong long-range interactions. Long-range interactions were then annotated using diffloop v1.10.0^81^ (default parameters with enhancer and promoter regions defined as below) to decide enhancer–enhancer loops, enhancer–promoter loops, and promoter–promoter loops. Here, promoters were defined as ± 2 kb regions surrounding gene transcription start sites. Enhancers were defined as identified H3K27Ac binding regions except those located at promoter regions.

### Pathway analysis

Pathway enrichment analysis was performed with gene names using DAVID version v6.8^82^. Selected KEGG pathways that have *P*-value less than 0.05 were reported

### Statistics and reproducibility

Statistics were done as stated in the figure legends. No statistical method was used to predetermine sample size. Sample sizes were chosen based on similar studies in the relevant literature. Source data are provided as a Source Data file.

### Reporting summary

Further information on research design is available in the Nature Research Reporting Summary linked to this article.

## Supplementary information

Supplementary Information

Description of Additional Supplementary Files

Supplementary Data 1

Supplementary Data 2

Reporting summary

## Data Availability

The H3K27ac ChIP-seq and HiChIP data were deposited in GEO, accession number “GSE136090”. The data can be visualized on human genome browser: http://epigenomegateway.wustl.edu/legacy/?genome=hg19&session=8jnrl5LWsd&statusId=843552307. KEGG database was obtained from DAVID version v6.8. Gene expression microarray data were download from GEO “GSE1880” for BC1, BC3, and BCBL1 cell lines. CTCF and SMC1 ChIP-seq data were downloaded from GEO, “GSE38411”. All other relevant data supporting the key findings of this study are available within the article and its Supplementary Information files or from the corresponding authors upon reasonable request. A reporting summary for this Article is available as a Supplementary Information file. Source data are provided with this paper.

## Source data

Source Data

## References

[1] Boulanger E (2005). Prognostic factors and outcome of human herpesvirus 8-associated primary effusion lymphoma in patients with AIDS. J. Clin. Oncol..

[2] Godfrey A, Anderson J, Papanastasiou A, Takeuchi Y, Boshoff C (2005). Inhibiting primary effusion lymphoma by lentiviral vectors encoding short hairpin RNA. Blood.

[3] Wies E (2008). The viral interferon-regulatory factor-3 is required for the survival of KSHV-infected primary effusion lymphoma cells. Blood.

[4] Shimada K, Hayakawa F, Kiyoi H (2018). Biology and management of primary effusion lymphoma. Blood.

[5] Guasparri I, Keller SA, Cesarman E (2004). KSHV vFLIP is essential for the survival of infected lymphoma cells. J. Exp. Med..

[6] Laherty CD, Hu HM, Opipari AW, Wang F, Dixit VM (1992). The Epstein-Barr virus LMP1 gene product induces A20 zinc finger protein expression by activating nuclear factor kappa B. J. Biol. Chem..

[7] Sun Q, Zachariah S, Chaudhary PM (2003). The human herpes virus 8-encoded viral FLICE-inhibitory protein induces cellular transformation via NF-kappa B activation. J. Biol. Chem..

[8] Trivedi P (2004). Infection of HHV-8+ primary effusion lymphoma cells with a recombinant Epstein-Barr virus leads to restricted EBV latency, altered phenotype, and increased tumorigenicity without affecting TCL1 expression. Blood.

[9] McHugh D (2017). Persistent KSHV infection increases EBV-associated tumor formation in vivo via enhanced EBV lytic gene expression. Cell Host Microbe.

[10] Bigi R (2018). Epstein-Barr virus enhances genome maintenance of Kaposi sarcoma-associated herpesvirus. Proc. Natl. Acad. Sci. USA.

[11] Roy D, Sin SH, Damania B, Dittmer DP (2011). Tumor suppressor genes FHIT and WWOX are deleted in primary effusion lymphoma (PEL) cell lines. Blood.

[12] Fan W (2005). Distinct subsets of primary effusion lymphoma can be identified based on their cellular gene expression profile and viral association. J. Virol..

[13] Manzano M (2018). Gene essentiality landscape and druggable oncogenic dependencies in herpesviral primary effusion lymphoma. Nat. Commun..

[14] Jiang S (2017). The Epstein-Barr virus regulome in lymphoblastoid cells. Cell Host Microbe.

[15] Maass PG, Barutcu AR, Rinn JL (2019). Interchromosomal interactions: a genomic love story of kissing chromosomes. J. Cell Biol..

[16] Mumbach MR (2016). HiChIP: efficient and sensitive analysis of protein-directed genome architecture. Nat. Meth..

[17] Mansour MR (2014). Oncogene regulation. An oncogenic super-enhancer formed through somatic mutation of a noncoding intergenic element. Science.

[18] Schmidt SC (2015). Epstein-Barr virus nuclear antigen 3A partially coincides with EBNA3C genome-wide and is tethered to DNA through BATF complexes. Proc. Natl. Acad. Sci. USA.

[19] Zhao B (2011). Epstein-Barr virus exploits intrinsic B-lymphocyte transcription programs to achieve immortal cell growth. Proc. Natl. Acad. Sci. USA.

[20] Zhao B (2014). The NF-kappa B genomic landscape in lymphoblastoid B cells. Cell Rep..

[21] Zhou H (2015). Epstein-Barr virus oncoprotein super-enhancers control B cell growth. Cell Host Microbe.

[22] Warburton A (2018). HPV integration hijacks and multimerizes a cellular enhancer to generate a viral-cellular super-enhancer that drives high viral oncogene expression. PLoS Genet.

[23] Yashiro-Ohtani Y (2014). Long-range enhancer activity determines Myc sensitivity to Notch inhibitors in T cell leukemia. Proc. Natl. Acad. Sci. USA.

[24] Ernst J (2011). Mapping and analysis of chromatin state dynamics in nine human cell types. Nature.

[25] Flavahan WA, Gaskell E, Bernstein BE (2017). Epigenetic plasticity and the hallmarks of cancer.. Science.

[26] Toth Z (2016). LANA-mediated recruitment of host polycomb repressive complexes onto the KSHV genome during de novo infection. PLoS Pathog..

[27] Toth Z (2013). Biphasic euchromatin-to-heterochromatin transition on the KSHV genome following de novo infection. PLoS Pathog..

[28] Hu J (2014). LANA binds to multiple active viral and cellular promoters and associates with the H3K4methyltransferase hSET1 complex. PLoS Pathog..

[29] Gunther T, Schreiner S, Dobner T, Tessmer U, Grundhoff A (2014). Influence of ND10 components on epigenetic determinants of early KSHV latency establishment. PLoS Pathog..

[30] Lu F (2012). Identification of host-chromosome binding sites and candidate gene targets for Kaposi’s sarcoma-associated herpesvirus LANA. J. Virol..

[31] Chen HS, Wikramasinghe P, Showe L, Lieberman PM (2012). Cohesins repress Kaposi’s sarcoma-associated herpesvirus immediate early gene transcription during latency. J. Virol..

[32] Whyte WA (2013). Master transcription factors and mediator establish super-enhancers at key cell identity genes. Cell.

[33] Servant N (2015). HiC-Pro: an optimized and flexible pipeline for Hi-C data processing. Genome Biol..

[34] Lareau CA, Aryee MJ (2018). hichipper: a preprocessing pipeline for calling DNA loops from HiChIP data. Nat. Methods.

[35] Quentmeier H (2019). The LL-100 panel: 100 cell lines for blood cancer studies. Sci. Rep..

[36] Belletti D (2016). PEGylated siRNA lipoplexes for silencing of BLIMP-1 in primary effusion lymphoma: in vitro evidences of antitumoral activity. Eur. J. Pharm. Biopharm..

[37] Riva G (2015). Antineoplastic effects of liposomal short interfering RNA treatment targeting BLIMP1/PRDM1 in primary effusion lymphoma. Haematologica.

[38] Ma Y (2017). CRISPR/Cas9 screens reveal Epstein-Barr virus-transformed B cell host dependency factors. Cell Host Microbe.

[39] Minamino K (2012). IRF-2 regulates B-cell proliferation and antibody production through distinct mechanisms. Int. Immunol..

[40] Thomas MD, Kremer CS, Ravichandran KS, Rajewsky K, Bender TP (2005). c-Myb is critical for B cell development and maintenance of follicular B cells. Immunity.

[41] Pfeffer SR, Yang CH, Pfeffer LM (2015). The role of miR-21 in cancer. Drug Dev. Res..

[42] Qin Z, Peruzzi F, Reiss K, Dai L (2014). Role of host microRNAs in Kaposi’s sarcoma-associated herpesvirus pathogenesis. Viruses.

[43] Tsai YH (2009). The M type K15 protein of Kaposi’s sarcoma-associated herpesvirus regulates microRNA expression via its SH2-binding motif to induce cell migration and invasion. J. Virol..

[44] Chapuy B (2013). Discovery and characterization of super-enhancer-associated dependencies in diffuse large B cell lymphoma. Cancer Cell.

[45] Liu T (2014). Use model-based analysis of ChIP-Seq (MACS) to analyze short reads generated by sequencing protein-DNA interactions in embryonic stem cells. Meth. Mol. Biol..

[46] Heinz S (2010). Simple combinations of lineage-determining transcription factors prime cis-regulatory elements required for macrophage and B cell identities. Mol. Cell.

[47] Belle I, Zhuang Y (2014). E proteins in lymphocyte development and lymphoid diseases. Curr. Top. Dev. Biol..

[48] Wang, C. et al. TAF family proteins and MEF2C are essential for Epstein-Barr virus super-enhancer activity. *J. Virol.* **93**, 10.1128/JVI.00513-19 (2019). 10.1128/JVI.00513-19 PMC6675876 31167905

[49] Jiang S (2014). Epstein-Barr virus nuclear antigen 3C binds to BATF/IRF4 or SPI1/IRF4 composite sites and recruits Sin3A to repress CDKN2A. Proc. Natl. Acad. Sci. USA.

[50] Arguello M (2003). Disruption of the B-cell specific transcriptional program in HHV-8 associated primary effusion lymphoma cell lines. Oncogene.

[51] Care MA (2014). SPIB and BATF provide alternate determinants of IRF4 occupancy in diffuse large B-cell lymphoma linked to disease heterogeneity. Nucleic Acids Res..

[52] Krappmann D, Vincendeau M (2016). Mechanisms of NF-kappaB deregulation in lymphoid malignancies. Semin. Cancer Biol..

[53] Matta H, Chaudhary PM (2004). Activation of alternative NF-kappa B pathway by human herpes virus 8-encoded Fas-associated death domain-like IL-1 beta-converting enzyme inhibitory protein (vFLIP). Proc. Natl. Acad. Sci. USA.

[54] An FQ (2005). The latency-associated nuclear antigen of Kaposi’s sarcoma-associated herpesvirus modulates cellular gene expression and protects lymphoid cells from p16 INK4A-induced cell cycle arrest. J. Biol. Chem..

[55] Gopalakrishnan R, Matta H, Tolani B, Triche T, Chaudhary PM (2016). Immunomodulatory drugs target IKZF1-IRF4-MYC axis in primary effusion lymphoma in a cereblon-dependent manner and display synergistic cytotoxicity with BRD4 inhibitors. Oncogene.

[56] Robson MI, Ringel AR, Mundlos S (2019). Regulatory landscaping: how enhancer-promoter communication is sculpted in 3D. Mol. Cell.

[57] Ryan RJH (2017). A B cell regulome links notch to downstream oncogenic pathways in small B cell lymphomas. Cell Rep..

[58] Ryan RJ (2015). Detection of enhancer-associated rearrangements reveals mechanisms of oncogene dysregulation in B-cell lymphoma. Cancer Disco..

[59] Yeo NC (2018). An enhanced CRISPR repressor for targeted mammalian gene regulation. Nat. Methods.

[60] Wong JP (2019). Kinome profiling of non-Hodgkin lymphoma identifies Tyro3 as a therapeutic target in primary effusion lymphoma. Proc. Natl. Acad. Sci. USA.

[61] Wang Y (2015). CDK7-dependent transcriptional addiction in triple-negative breast cancer. Cell.

[62] Sabari BR (2018). Coactivator condensation at super-enhancers links phase separation and gene control.. Science.

[63] Tolani B, Gopalakrishnan R, Punj V, Matta H, Chaudhary PM (2014). Targeting Myc in KSHV-associated primary effusion lymphoma with BET bromodomain inhibitors. Oncogene.

[64] Chen HS (2017). BET-inhibitors disrupt Rad21-dependent conformational control of KSHV latency. PLoS Pathog..

[65] Brown JD (2014). NF-kappaB directs dynamic super enhancer formation in inflammation and atherogenesis. Mol. Cell.

[66] Park A (2020). Global epigenomic analysis of KSHV-infected primary effusion lymphoma identifies functional MYC superenhancers and enhancer RNAs. Proc. Natl. Acad. Sci. USA.

[67] Klein U (2003). Gene expression profile analysis of AIDS-related primary effusion lymphoma (PEL) suggests a plasmablastic derivation and identifies PEL-specific transcripts. Blood.

[68] Basso K, Dalla-Favera R (2012). Roles of BCL6 in normal and transformed germinal center B cells. Immunol. Rev..

[69] Manzano M (2020). Kaposi’s sarcoma-associated Herpesvirus drives a super-enhancer-mediated survival gene expression program in primary effusion lymphoma.. mBio.

[70] Elabd S, Meroni G, Blattner C (2016). TRIMming p53’s anticancer activity. Oncogene.

[71] Skalsky RL (2007). Kaposi’s sarcoma-associated herpesvirus encodes an ortholog of miR-155. J. Virol..

[72] Irizarry RA (2003). Exploration, normalization, and summaries of high density oligonucleotide array probe level data. Biostatistics.

[73] Langmead B, Salzberg SL (2012). Fast gapped-read alignment with Bowtie 2. Nat. Methods.

[74] Zhang Y (2008). Model-based analysis of ChIP-Seq (MACS). Genome Biol..

[75] Landt SG (2012). ChIP-seq guidelines and practices of the ENCODE and modENCODE consortia. Genome Res..

[76] Li Q, Huang BJH, Bickel PJ (2011). Measuring reproducibility of high-throughput experiments. Ann. Appl. Stat..

[77] Amemiya HM, Kundaje A, Boyle AP (2019). The ENCODE blacklist: identification of problematic regions of the genome. Sci. Rep..

[78] Ross-Innes CS (2012). Differential oestrogen receptor binding is associated with clinical outcome in breast cancer. Nature.

[79] Love MI, Huber W, Anders S (2014). Moderated estimation of fold change and dispersion for RNA-seq data with DESeq2. Genome Biol..

[80] Zhou X (2013). Exploring long-range genome interactions using the WashU Epigenome Browser. Nat. Methods.

[81] Lareau CA, Aryee MJ (2018). Diffloop: a computational framework for identifying and analyzing differential DNA loops from sequencing data. Bioinformatics.

[82] Huang da W, Sherman BT, Lempicki RA (2009). Systematic and integrative analysis of large gene lists using DAVID bioinformatics resources. Nat. Protoc..

