# Supplementary material for: Primary effusion lymphoma enhancer connectome links super-enhancers to dependency factors
Source: Nat Commun. 2020 Dec 9;11:6318. doi: 10.1038/s41467-020-20136-w (PMC7726151; doi:10.1038/s41467-020-20136-w)
Supplement: Supplementary file 1 — Supplementary Information [file 41467_2020_20136_MOESM1_ESM.pdf]

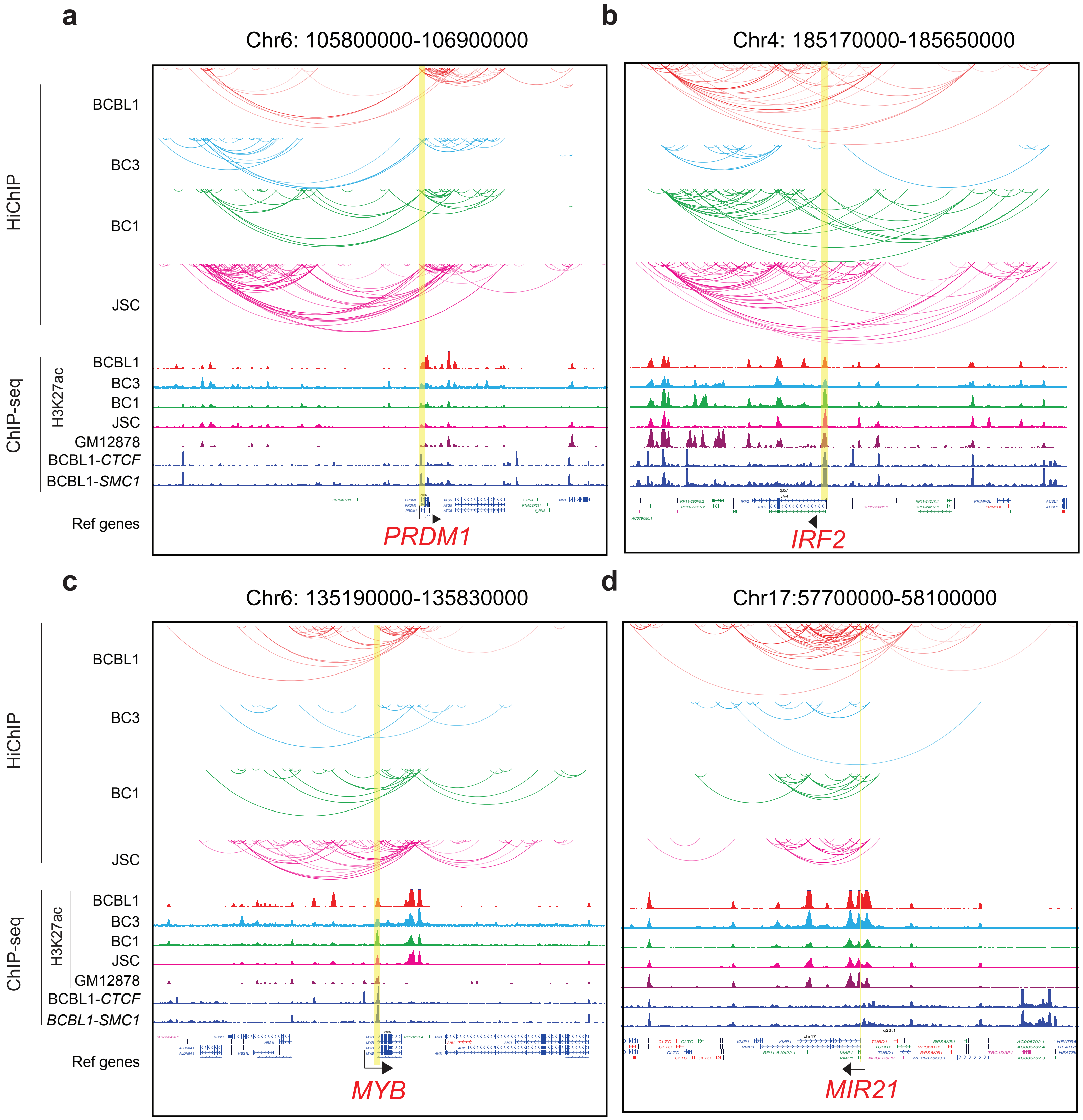

Supplementary Figure 1. Genomic interactions at PRDM1, IRF2, MYB, and MIR21 loci identified by H3K27ac HiChIP. H3K27ac HiChIPs were done in BCBL1, BC3, BC1, and JSC cells. Each curved line represents a significant genomic interaction. Black arrows indicate the direction of transcription. Yellow vertical bars represent promoters. a PRDM1, b IRF2, c MYB, and d MIR21.

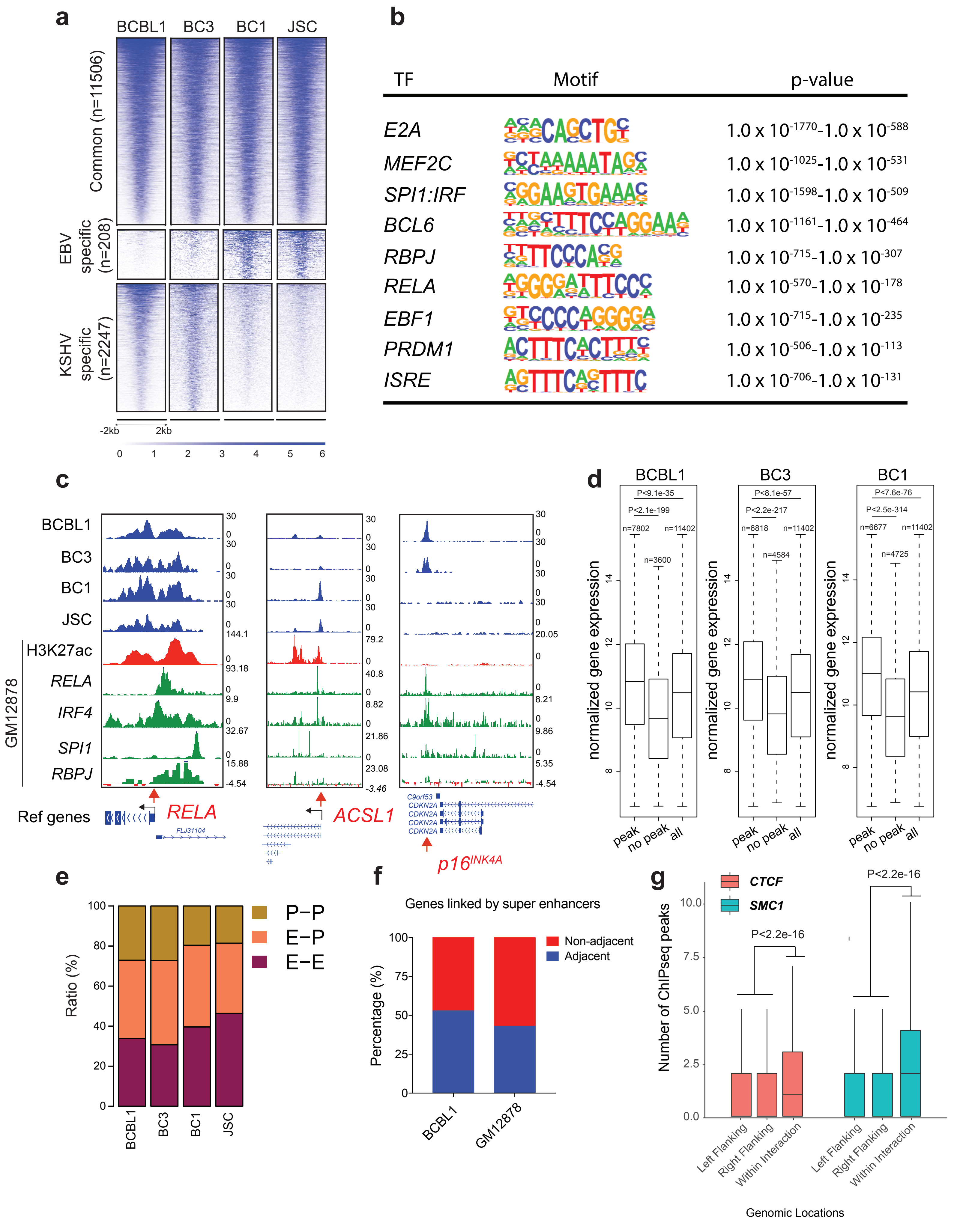

a
BCBL1
BC3
BC1
JSC
Common (n=11506)
EBV specific (n=208)
KSHV specific (n=2247)
-2kb
2kb
0
1
2
3
4
5
6
b
TF
Motif
p-value
E2A
1.0 x 10-1770-1.0 x 10-588
MEF2C
1.0 x 10-1025-1.0 x 10-531
SPI1:IRF
1.0 x 10-1598-1.0 x 10-509
BCL6
1.0 x 10-1161-1.0 x 10-464
RBPJ
1.0 x 10-715-1.0 x 10-307
RELA
1.0 x 10-570-1.0 x 10-178
EBF1
1.0 x 10-715-1.0 x 10-235
PRDM1
1.0 x 10-506-1.0 x 10-113
ISRE
1.0 x 10-706-1.0 x 10-131
c
BCBL1
BC3
BC1
JSC
GM12878
H3K27ac
RELA
IRF4
SPI1
RBPJ
Ref genes
RELA
FLJ31104
ACSL1
C9orf53
CDKN2A
p16INK4A
d
BCBL1
BC3
BC1
P<9.1e-35
P<2.1e-199
n=7802
n=3600
n=11402
P<8.1e-57
P<2.2e-217
n=6818
n=4584
n=11402
P<7.6e-76
P<2.5e-314
n=6677
n=4725
n=11402
normalized gene expression
peak
no peak
all
e
Ratio (%)
P−P
E−P
E−E
BCBL1
BC3
BC1
JSC
f
Genes linked by super enhancers
Percentage (%)
Non-adjacent
Adjacent
BCBL1
GM12878
g
CTCF
SMC1
P<2.2e-16
P<2.2e-16
Number of ChIPseq peaks
Left Flanking
Right Flanking
Within Interaction
Genomic Locations

Supplementary Figure 2. PEL enhancer landscapes, enhancer associated genes expression, TF binding and interaction partners. H3K27ac ChIP-seqs were done in PEL cell lines BC1, JSC, BC3 and BCBL1. Significant peaks were called by MACS. a Heatmap of H3K27ac ChIP-seq peaks common for all PEL cell lines, common peaks in BC1 and JSC but lack signal in BC3 and BCBL1, common peaks in BC3 and BCBL1 but lack signal in BC1 and JSC. b Motifs significantly enriched in the PEL H3K27ac ChIP-seq peaks identified by HOMER. c GM12878 RELA, IRF4, SPI1, and RBPJ signals at RELA, ASCL1, and p16INK4A. d Expression of PEL enhancer associated genes in BCBL1, BC3, and BC1 cells correlated with H3K27ac ChIP-seq (two-sided t-test with unequal variance was applied). Genes near H3K27ac peaks are expressed at significantly higher than genes without. e H3K27ac HiChIPs were grouped into enhancer-enhancer, enhancer-promoter, promoter-promoter interactions. Promoter is defined by +/- 2 kb from the TSS. f. Enhancers and their direct targets were linked by HiChIP. Enhancer-gene links are categorized into two types, links between enhancers and immediate adjacent genes and links skipping the immediate adjacent genes. g. CTCF and SMC1 binding signals within SE-target HiChIP loops and their neighboring control regions are shown for BCBL1 cells ($n=3502$, two-sided paired Wilcox test is performed there with $p < 2.2e-16$). HiChIP interaction regions were first merged for overlapping interactions. Neighboring control regions are calculated as the same size of regions from the left and right adjacent flanking of the HiChIP interaction regions. CTCF and SMC1 peaks was called using ChIP-seq data downloaded from GEO, GSE38411. Detail of boxplot plots in d and g: center value is the medium; upper and lower bounds of boxes are upper and lower quartile, respectively; whiskers extend by 1.5*(upper quartile - lower quartile) or the extreme value of all the data (minimum/maximum), whichever comes first.

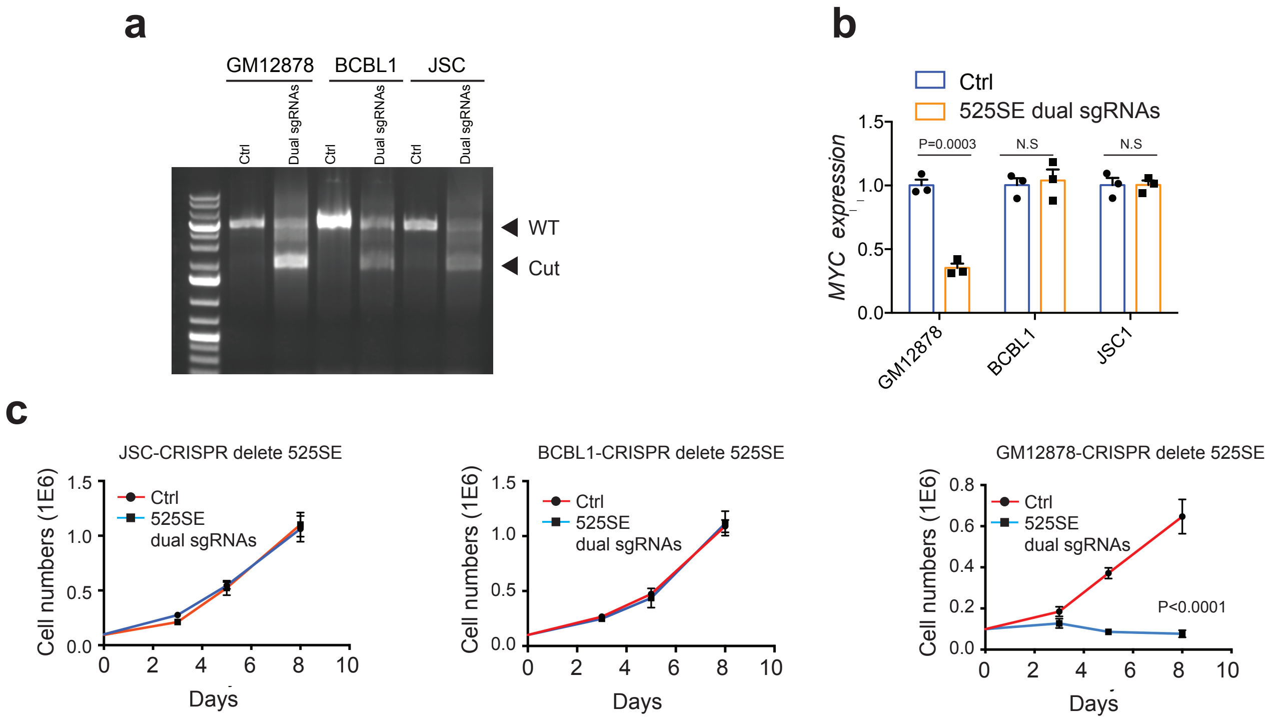

Supplementary Figure 3. Deletion of GM12878 MYC SE does not affect MYC expression in PEL and PEL growth. a Lentiviruses expressing dual gRNAs targeting the edges of MYC 525 SE or control gRNA were used to transduce GM12878, BCBL1, and JSC cells stably expressing CAS9. After puromycin selection, genomic DNAs were extracted from these cells. SE DNAs were PCR amplified and visualized by agarose gel electrophoresis. (n=3, independent experiments with similar results). b MYC expression by qRT-PCR following dual gRNA CRISPR deletion. (n=3, independent experiments. ns non-significant). A two-tailed unpaired t-test was used for statistical analysis. c Cell growth following dual gRNA CRISPR deletion (n=4, independent experiments. ns non-significant). Two-way analysis of variance (ANOVA) followed by Bonferroni multiple comparison analysis was used for analysis. The error bars indicate the SEM for the averages across the multiple experiments.

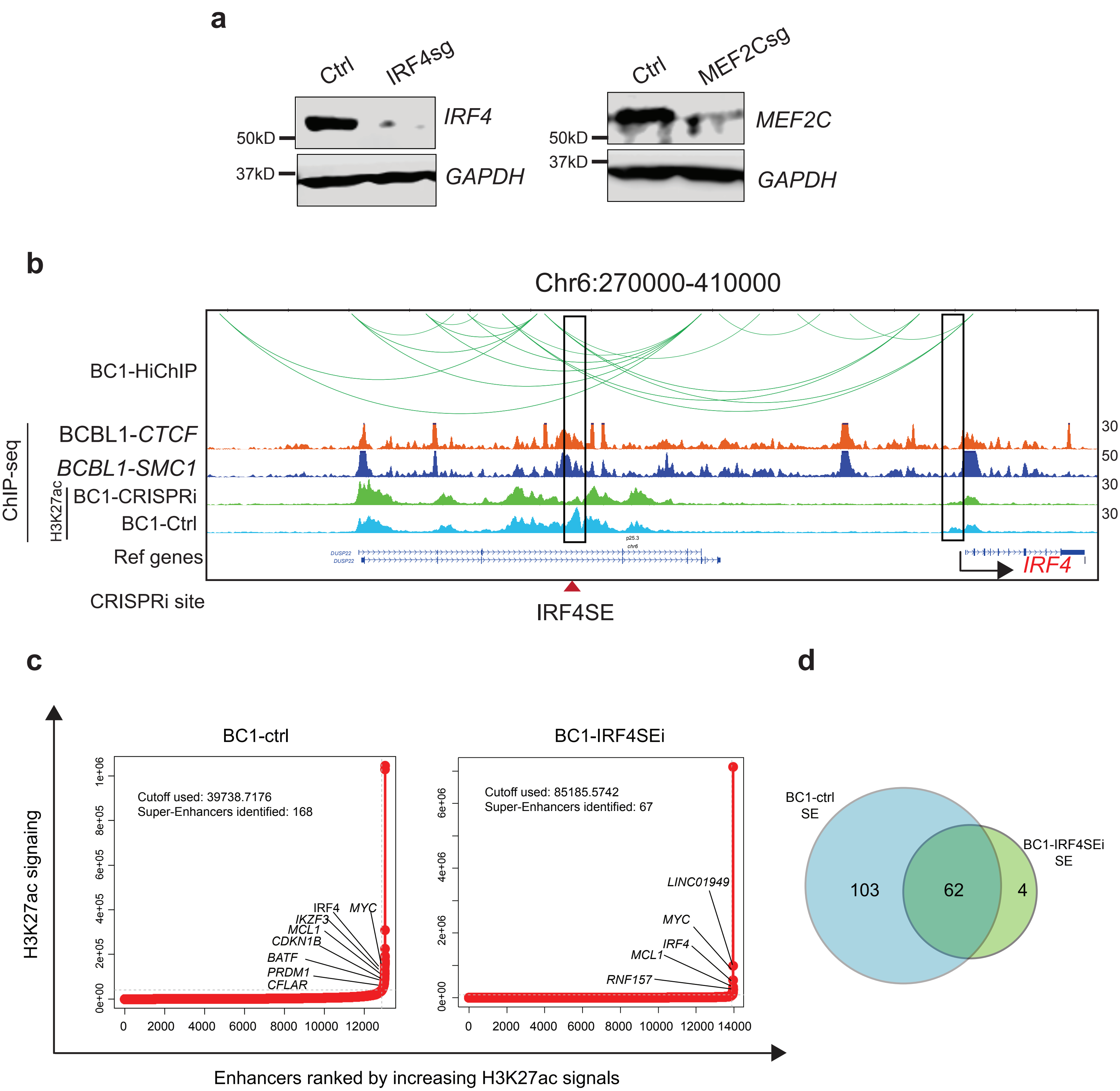

Supplementary Figure 4. IRF4 and MEF2C CRISPR knock out in BCBL1. a Lentiviruses expressing sgRNAs targeting IRF4 and MEF2C were used to transduce PEL cells stably expressing CAS9. After puromycin selection, cells were harvested. IRF4 and MEF2C expression was examined by Western blotting. GAPDH was used as loading control. (n=3, independent experiments with similar results). b H3K27ac ChIP-seq signals at the IRF4 locus. PEL cell treated with IRF4 SE CRISPRi with sgRNA2 or control sgRNA. H3K27ac ChIP-seqs were done to determine the effect of CRISPRi on PEL cell enhancer landscape. c. SEs in control or IRF4 SE sgRNA treated cells. d SE overlap between control or IRF4 SE sgRNA treated cells.

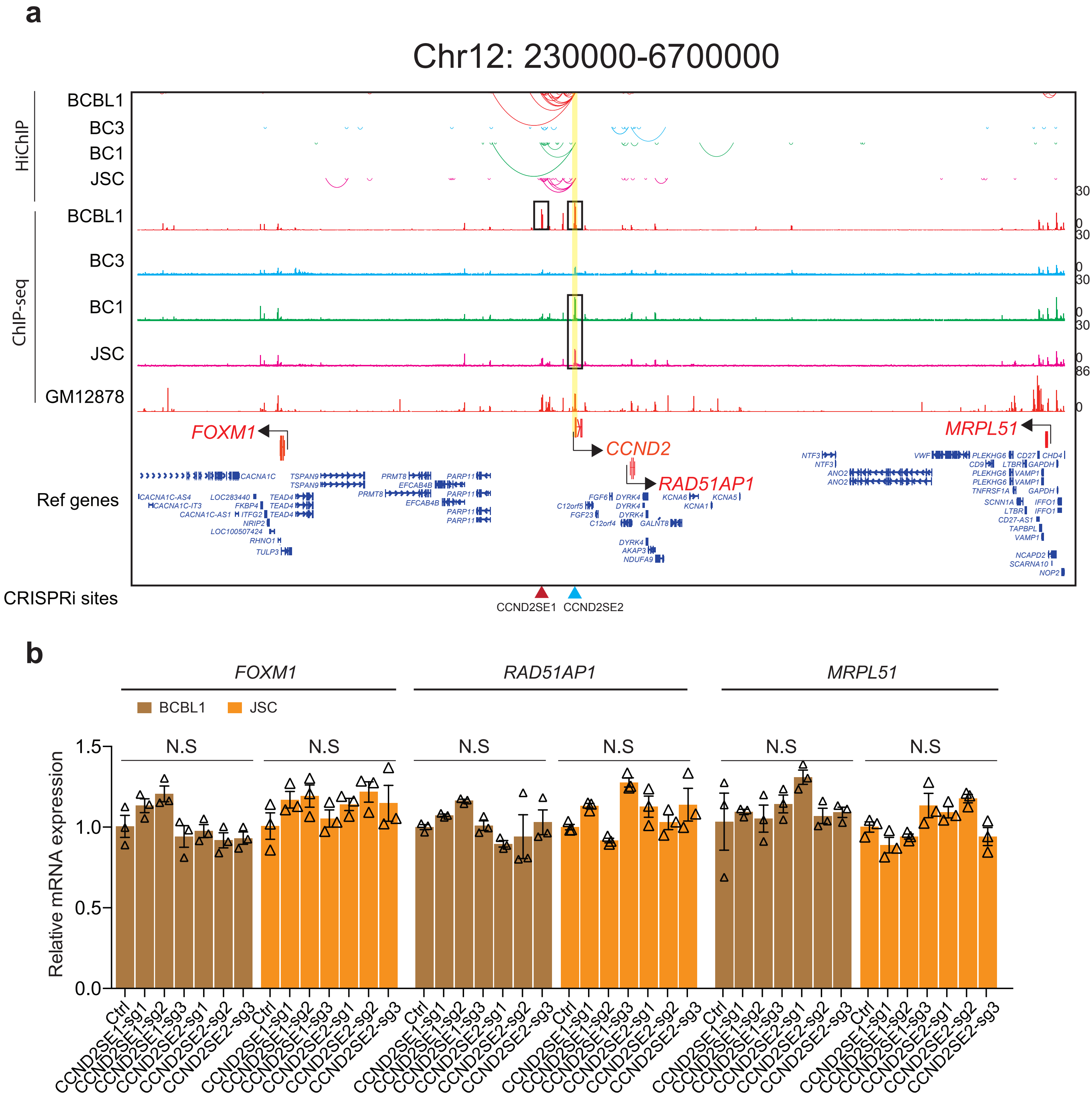

Supplementary Figure 5. CRISPRi inhibition of CCND2 SE does not affect the expression of neighboring genes. a HiChIP links at the CCND2 loci. b Expression of FOXM1, RAD51AP1, and MRLP51 followed by CCND2 CRISPRi inhibition. The levels of non-targeting sgRNA were set to 1. (n=3, independent experiments. ns non-significant). A two-tailed unpaired t-test was used for statistical analysis. The error bars indicate the SEM for the averages across the multiple experiments.

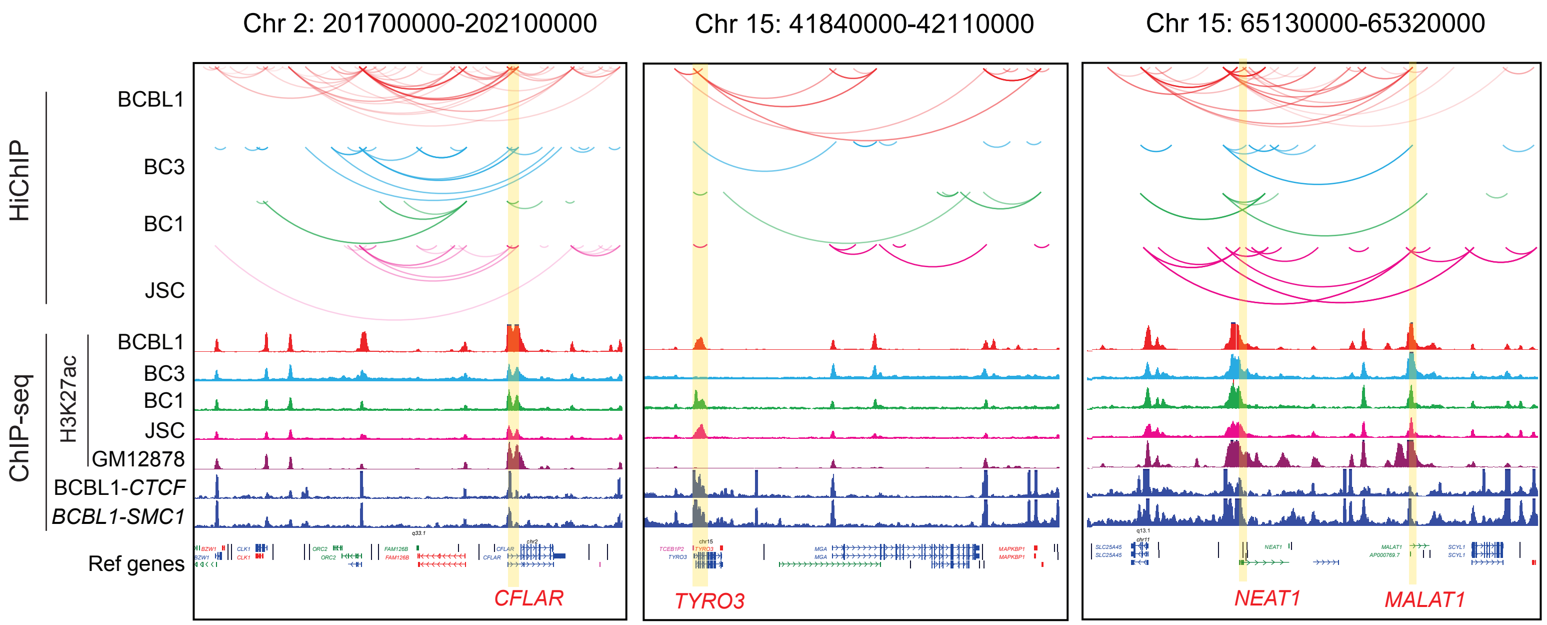

Supplementary Figure 6. HiChIP links SEs to CFLAR, TYRO3, NEAT1, and MALAT1. HiChIPs are shown on the top with each curved link indicates a significant interaction. ChIP-seq tracks are shown below. Black boxes indicate SEs.

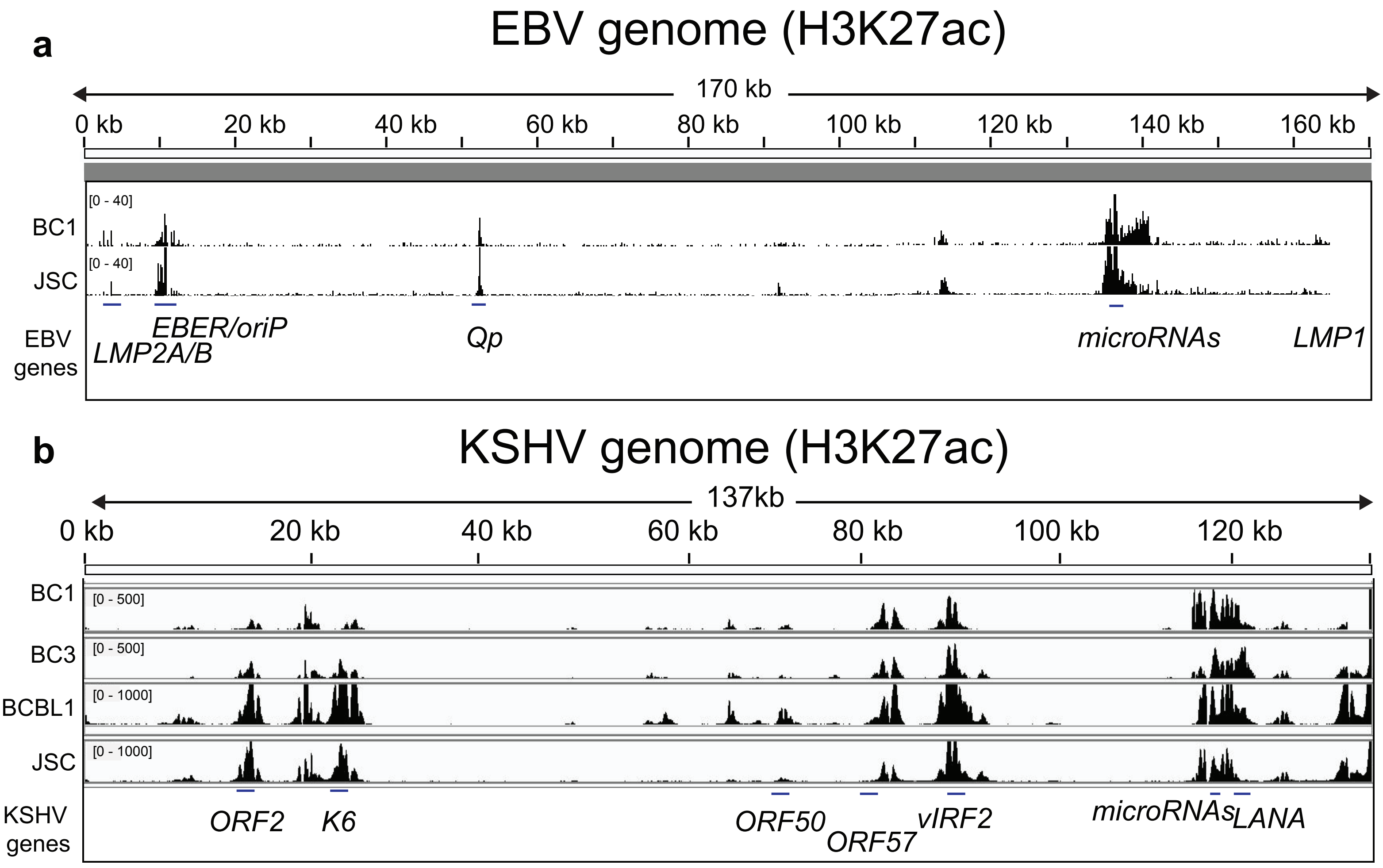

Supplementary Figure 7. KSHV and EBV genome H3K27ac landscapes. a PEL H3K27ac ChIP-seq reads were mapped to the EBV genome and visualized using IGV. EBV genome annotation is shown under the ChIP-seq tracks b PEL H3K27ac ChIP-seq reads were mapped to the KSHV genome and visualized in IGV. KSHV genome annotation is shown under the ChIP-seq tracks.
