# Supplementary material for: Primary effusion lymphoma enhancer connectome links super-enhancers to dependency factors
Source: Nat Commun. 2020 Dec 9;11:6318. doi: 10.1038/s41467-020-20136-w (PMC7726151; doi:10.1038/s41467-020-20136-w)
Supplement: Supplementary file 2 — Description of Additional Supplementary Files [file 41467_2020_20136_MOESM2_ESM.pdf]

Description of Additional Supplementary Files

Supplementary Data 1: SEs were linked to their target genes by H3K27ac HiChIP for each of the PEL cell line. The linked genes for each cell line are listed in the dataset.

Supplementary Data 2: Primers used in the paper.
