# Supplementary material for: Primary effusion lymphoma enhancer connectome links super-enhancers to dependency factors
Source: Nat Commun. 2020 Dec 9;11:6318. doi: 10.1038/s41467-020-20136-w (PMC7726151; doi:10.1038/s41467-020-20136-w)
Supplement: Supplementary file 5 — Reporting summary [file 41467_2020_20136_MOESM5_ESM.pdf]

nature research

Corresponding author(s): Bo Zhao

Last updated by author(s): Oct 15, 2020

# Reporting Summary

Nature Research wishes to improve the reproducibility of the work that we publish. This form provides structure for consistency and transparency in reporting. For further information on Nature Research policies, see Authors & Referees and the Editorial Policy Checklist .

## Statistics

For all statistical analyses, confirm that the following items are present in the figure legend, table legend, main text, or Methods section.

| n/a | Confirmed | |
|---|---|---|
| ☐ | ☒ | The exact sample size (*n*) for each experimental group/condition, given as a discrete number and unit of measurement |
| ☐ | ☒ | A statement on whether measurements were taken from distinct samples or whether the same sample was measured repeatedly |
| ☐ | ☒ | The statistical test(s) used AND whether they are one- or two-sided<br>*Only common tests should be described solely by name; describe more complex techniques in the Methods section.* |
| ☒ | ☐ | A description of all covariates tested |
| ☒ | ☐ | A description of any assumptions or corrections, such as tests of normality and adjustment for multiple comparisons |
| ☐ | ☒ | A full description of the statistical parameters including central tendency (e.g. means) or other basic estimates (e.g. regression coefficient) AND variation (e.g. standard deviation) or associated estimates of uncertainty (e.g. confidence intervals) |
| ☐ | ☒ | For null hypothesis testing, the test statistic (e.g. *F*, *t*, *r*) with confidence intervals, effect sizes, degrees of freedom and *P* value noted<br>*Give P values as exact values whenever suitable.* |
| ☒ | ☐ | For Bayesian analysis, information on the choice of priors and Markov chain Monte Carlo settings |
| ☒ | ☐ | For hierarchical and complex designs, identification of the appropriate level for tests and full reporting of outcomes |
| ☒ | ☐ | Estimates of effect sizes (e.g. Cohen's *d*, Pearson's *r*), indicating how they were calculated |

*Our web collection on statistics for biologists contains articles on many of the points above.*

## Software and code

Policy information about availability of computer code

| | |
|---|---|
| Data collection | No software was used for data collection |
| Data analysis | FastQC (https://www.bioinformatics.babraham.ac.uk/projects/fastqc) was performed on both ChIP-seq and HiChIP sequencing reads to ensure sequencing experiments have no considerable flaws such as heavy GC bias and PCR artifacts. ChIP-seq reads were aligned to human (hg19) and EBV (Akata) genomes using Bowtie2 v2.2.3. ChIP-seq peaks were called using MACS v2.1.0 The HOMER program was then used to detected motifs in ChIP-seq peaks. Genome-wide ChIP-seq coverage were normalized with size factors which were determined using DiffBind combined with DESeq2. Super-enhancers were called with ROSE v1.0.0 under default settings using IDR reported H3K27ac peaks. HiChIP paired-end reads (17-27 million reads for each sample) were mapped using HiC-Pro v2.11.1 (default settings with LIGATION_SITE set as GATCGATC for MboI) and significant loops identified with hichipper v0.7.5 (default settings except parameter --skip-diffloop was set) .Long range interactions were annotated using diffloop v1.10.0 |

For manuscripts utilizing custom algorithms or software that are central to the research but not yet described in published literature, software must be made available to editors/reviewers. We strongly encourage code deposition in a community repository (e.g. GitHub). See the Nature Research guidelines for submitting code & software for further information.

## Data

Policy information about availability of data

All manuscripts must include a data availability statement. This statement should provide the following information, where applicable:

- Accession codes, unique identifiers, or web links for publicly available datasets
- A list of figures that have associated raw data
- A description of any restrictions on data availability

The H3K27ac ChIP-seq and HiChIP data were deposited in GEO, accession number GSE136090 (https://www.ncbi.nlm.nih.gov/geo/query/acc.cgi?acc=GSE136090). The data can be visualized on human genome browser: http://epigenomegateway.wustl.edu/legacy/?genome=hg19&session=8jnrl5LWsd&statusId=843552307. KEGG database was obtained from DAVID version v6.8. Gene expression microarray data was download from GEO (GSE1880) for BC1, BC3, BCBL1 cell lines. CTCF

and SMC1 ChIP-seq data was downloaded from GEO, GSE38411. The source data underlying Figs 2b,c,e,f,g,h; 3b,c,d,f,h,I; 4b,c,e,f,h,i; 5a-c and Supplementary Figs 2d,2e,3b,3c,4a, 4c and 5b are provided as a Source Data file. Primers used in this study are provided in Supplementary Data 2.

# Field-specific reporting

Please select the one below that is the best fit for your research. If you are not sure, read the appropriate sections before making your selection.

[x] Life sciences [ ] Behavioural & social sciences [ ] Ecological, evolutionary & environmental sciences

For a reference copy of the document with all sections, see nature.com/documents/nr-reporting-summary-flat.pdf

# Life sciences study design

All studies must disclose on these points even when the disclosure is negative.

| | |
|---|---|
| Sample size | Sample size was determined based on cell lines available for our study. In this manuscript, we used four PEL cell lines including two KSHV positive two KSHV and EBV dual positive PEL cell lines. References include: Guasparri et al. J Exp Med, 2014, Bigi et al. Proc Natl Acad Sci U S A 2018, Wies et al. Blood 2008. |
| Data exclusions | No data were excluded from the analysis |
| Replication | HiChIP was performed with two biological replicates and all data were reported. 3-6 independent replicates were performed as indicated in figure legends with a frequent of 7-10 days per replicate. |
| Randomization | We cultured cells at multiple plates, cells at similar concentration were randomly selected for various treatment. |
| Blinding | We did not use group allocation, so Blinding is not applicable in our experiement |

# Reporting for specific materials, systems and methods

We require information from authors about some types of materials, experimental systems and methods used in many studies. Here, indicate whether each material, system or method listed is relevant to your study. If you are not sure if a list item applies to your research, read the appropriate section before selecting a response.

## Materials & experimental systems

| n/a | Involved in the study | |
|---|---|---|
| [ ] | [x] | Antibodies |
| [ ] | [x] | Eukaryotic cell lines |
| [x] | [ ] | Palaeontology |
| [x] | [ ] | Animals and other organisms |
| [x] | [ ] | Human research participants |
| [x] | [ ] | Clinical data |

## Methods

| n/a | Involved in the study | |
|---|---|---|
| [ ] | [x] | ChIP-seq |
| [x] | [ ] | Flow cytometry |
| [x] | [ ] | MRI-based neuroimaging |

## Antibodies

| | |
|---|---|
| Antibodies used | Name: H3K27Ac Antibody, Cat: ab4729, Supplier: Abcam<br>Name: GAPDH Antibody, Cat:ab9485, Supplier: Abcam<br>Name: MYC Antibody, Cat: 13987, Supplier: Cell signaling technology<br>Name: MEF2C Antibody, Cat: 5030, Supplier: Cell signaling technology<br>Name: IRF4 Antibody, Cat: sc-377383, Supplier: Santa Cruze<br>Name: CAS9 antibody, Cat: MA1-201, Supplier: Thermo Fisher. |
| Validation | Name: H3K27Ac Antibody, Species reactivity: Mouse, Rat, Chicken, Cow, Human, Arabidopsis thaliana, Drosophila melanogaster, Monkey, Zebrafish, Plasmodium falciparum, Rice, Cyanidioschyzon merolae, Application: IHC-Fr, ICC/IF, WB, IHC-P, CHIPseq, ChIP/Chip, ChIP, PepArr Data Link:https://www.abcam.com/histone-h3-acetyl-k27-antibody-chip-grade-ab4729.html.<br><br>Name: GAPDH Antibody,Species reactivity: Mouse, Rat, Chicken, Dog, Human, Saccharomyces cerevisiae, Xenopus laevis, Schizosaccharomyces pombe, African green monkey, Application: IHC-P, IP, ELISA, WB, IHC-Fr, ICC/IF, Flow Cyt Data Link: https://www.abcam.com/gapdh-antibody-loading-control-ab9485.html.<br><br>Name: MYC Antibody, Species reactivity: Human, Mouse, Rat, Monkey, Application: Western Blotting, Immunofluorescence (Immunocytochemistry) Flow Cytometry, Chromatin IP, Chromatin IP-seq. Data Link: https://www.cellsignal.com/products/primary-antibodies/c-myc-n-myc-d3n8f-rabbit-mab/13987 |

Name: MEF2C Antibody, Species reactivity: Human, Mouse, Application: Western Blotting, Immunoprecipitation, Immunofluorescence (Immunocytochemistry). Data Link: https://www.cellsignal.com/products/primary-antibodies/mef2c-d80c1-xp-rabbit-mab/5030?site-search-type=Products.

Name: IRF4 Antibody, Species reactivity: mouse, rat and human, Application: Western Blotting, Immunoprecipitation, Immunofluorescence, solid phase ELISA . Data Link: https://www.scbt.com/p/irf-4-antibody-e-7

Name: CAS9 antibody, Species reactivity: Bacteria, Application: Immunocytochemistry (ICC)

Immunofluorescence (IF), Western Blot (WB). Data Link: https://www.thermofisher.com/antibody/product/Cas9-Antibody-clone-7A9-3A3-Monoclonal/MA1-201

# Eukaryotic cell lines

Policy information about cell lines

| | |
|---|---|
| Cell line source(s) | BC1, BC3 and 293T cells were purchased from ATCC, BCBL1 was obtained from NIH AIDS Reagent program, JSC was obtained from Richard F Ambinder's lab |
| Authentication | None of the cell lines used were authenticated. |
| Mycoplasma contamination | All cell lines tested were negative for Mycoplasma contamination |
| Commonly misidentified lines (See ICLAC register) | No cell line is misidentified in this study |

# ChIP-seq

## Data deposition

- [x] Confirm that both raw and final processed data have been deposited in a public database such as GEO.
- [x] Confirm that you have deposited or provided access to graph files (e.g. BED files) for the called peaks.

| | |
|---|---|
| Data access links *May remain private before publication.* | https://www.ncbi.nlm.nih.gov/geo/query/acc.cgi?acc=GSE136090. The following secure token has been created to allow review of record GSE136090 while it remains in private status: mfufiecmthadjol. |
| Files in database submission | GSM4040941,GSM4040942,GSM4040943,GSM4040944,GSM4040945,GSM4040946,GSM4040947,GSM4040948,GSM4040949,GSM4040950,GSM4040951,GSM4040952. GSM4722031, GSM4722032, GSM4722033, GSM4722034, GSM4722035. Each dataset include raw sequencing data and processed data. For ChIP-seq, processed data is the peak list; for HiChIP, processed data is the long-range interactions. |
| Genome browser session (e.g. UCSC) | http://epigenomegateway.wustl.edu/legacy/?genome=hg19&session=8jnrl5LWsd&statusId=843552307 |

## Methodology

| | |
|---|---|
| Replicates | Two biological replicates are performed for each cell line on both ChIP-seq and HiChIP. replicate agreement is controlled using irreproducible discovery rate. |
| Sequencing depth | ChIP-seq was done using 75bp single-end sequencing with coverage for each cell type ranging from 17M to 80M total reads, among which over 95% are properly mapped. HiChIP was done using 75bp paired-end sequencing with total reads ranging from 69M to 108M for each replicate. The numbers of interaction reads aligned for each cell type range from 37M to 50M, among which over 31% on average are valid interactions. |
| Antibodies | H3K27Ac Antibody was used for ChIP-seq Cat: ab4729, Supplier: Abcam |
| Peak calling parameters | Peak calling was done using MACS2 using default parameters except we use q-value 0.99 to generate a flexible set of peaks, in order to compare the reproducible peaks between replicates using IDR tool. The final output of peaks for each cell type were reported based on idr<=0.02. |
| Data quality | Total numbers of peaks called by MACS2 for each replicate range from 63,179 to 377,879 with q-value 0.99. After reproducibility controlled based on IDR<0.02, total numbers of final peaks called for each cell type range from 21293 to 39803. |
| Software | ChIP-seq reads were aligned to human genome hg19 using Bowtie v2.2.3. ChIP-seq peaks were called on each replicate samples using MACS v2.1.0 by comparing ChIP to INPUT control. Peaks were merged from replicates in each cell line using IDR v2.0.3. HiChIP reads were aligned to human genome hg19 using HiC-Pro v2.11.1. All valid interaction read pairs were then analyzed with hichipper v0.7.5 for detection of significant long-range interactions. Interaction scores were normalized by the total number of valid read pairs. |
